# Understanding the Role of H‑Bonds in the Stability of Molecular Glue-Induced Ternary Complexes

**DOI:** 10.1021/acs.jcim.5c02718

**Published:** 2026-01-23

**Authors:** Patricia Blanco-Gabella, Varbina Ivanova, Álvaro Serrano-Morrás, Julian E. Fuchs, Jordi Juárez-Jiménez

**Affiliations:** † Departament de Farmàcia i Tecnologia Farmacèutica, i Fisicoquímica, Facultat de Farmàcia i Ciències de l’Alimentació, 16724 University of Barcelona, Joan XXIII 27-31, 08028 Barcelona, Spain; ‡ Institut de Química Teòrica i Computacional (IQTC), Facultat de Química i Física, Universitat de Barcelona (UB), C. Martí i Franqués, 1, 08028 Barcelona, Spain; § Institut de Biomedicina (IBUB), Facultat de Biologia, Universitat de Barcelona, Av. Diagonal 643, 08028 Barcelona, Spain; ∥ Boehringer Ingelheim RCV GmbH & Co. KG, Dr. Boehringer Gasse 5-11, 1121 Vienna, Austria

## Abstract

Protein–protein interaction (PPI) networks play a central role in many biological processes, and thus, the possibility of modulating them using small molecules offers several therapeutic opportunities. Molecular glues (MGs) are small molecules that bind to a PPI interface and stabilize the complex. Oftentimes, MGs show no measurable affinity for at least one of the proteins involved in the ternary complex, and the molecular bases for their action are not completely understood. We previously reported a significant correlation between protein–protein hydrogen bond robustness and the stability of the CRBN–CK1α complex induced by the antimyeloma drug lenalidomide. In this work, we demonstrate that this relationship is not unique for that system but rather represents a reproducible physicochemical phenomenon underlying the mechanism of action of chemically diverse MGs, including additional IMiDs and Fusicoccin A. Our results shed light on a vaguely understood phenomenon and pave the way for the development of new computational methods that enable the rational discovery of molecular glues.

## Graphical abstract

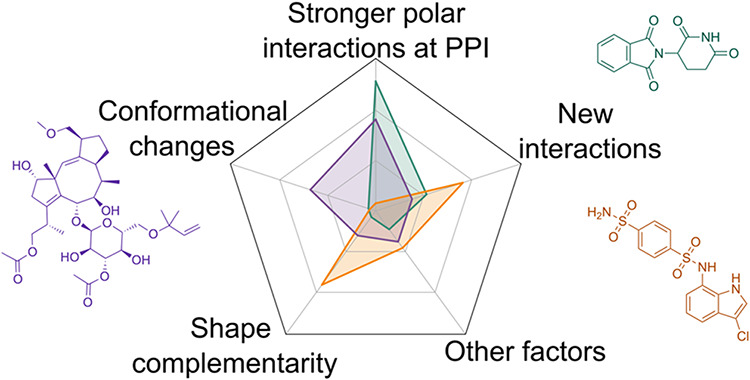

## Introduction

Cells are complex systems where countless protein–protein interactions (PPIs) occur simultaneously, forming dynamic regulatory networks that are essential to control many biological processes, such as cell signaling, homeostasis, and proliferation. As an example, anomalous PPIs have been linked to many diseases, such as cancer or Alzheimer's disease, and the modulation of PPIs has been deemed as a promising strategy for the treatment of refractory diseases.^1^ Therefore, the pharmacological regulation of this so-called interactome, estimated to account for more than 650,000 PPIs,^2^ offers tantalizing therapeutic opportunities. While blocking pre-existing protein interactions is a long-established strategy,^3–5^ the possibility of stabilizing PPIs has recently gained attention with the emergence of induced proximity pharmacology.^6^ This strategy extends beyond stabilizing experimentally characterized PPIs and aims to induce never-before-seen complexes by the use of the appropriate chemical probes.^7–11^ Among the several possible strategies that can promote the stabilization of PPIs, Cao et al. recently distinguished molecular glues (MGs) from other inducers of PPIs as a unique class of PPI-promoting molecules which extend the existing interaction interfaces without showing a detectable affinity toward at least one of the binding partners.^12,13^ From that definition, the conformational selection theory provides a useful theoretical framework to describe their mechanism of action (Figure 1). In the crowded intracellular space, at any given time, there would be thousands of high-energy, unstable protein–protein contacts, most of them too short-lived to be experimentally characterized. Some of these momentary contacts would involve labile interactions that can be stabilized by the presence of the appropriate MGs bound to one of the partners. However, the transient nature of these protein–protein complexes makes them essentially “invisible” to structural biology approaches, making it very difficult to anticipate the physicochemical features that an MG should contain to effectively stabilize any given contact. The ternary complexes, on the other hand, are commonly high-affinity, long-lived complexes that can modify the biological activity of one or both proteins involved and are suitable for characterization by biophysical techniques. The practical consequence of this mechanism of action is that, to date, MGs have been discovered rather serendipitously, oftentimes described phenotypically and characterized as an MG *a posteriori*, usually years or decades later.^14–17^ Nevertheless, their capacity to modulate transcription factors or IDPs makes them extremely relevant in the current drug discovery landscape. In addition, they typically present better ADMET profiles than other inducers of chemical proximity, which makes them more attractive for drug discovery programs. However, the lack of understanding of the underlying structural determinants governing the formation of MG-dependent ternary complexes has been a major obstacle in the rational development of MGs.^18^

**Figure 1:**
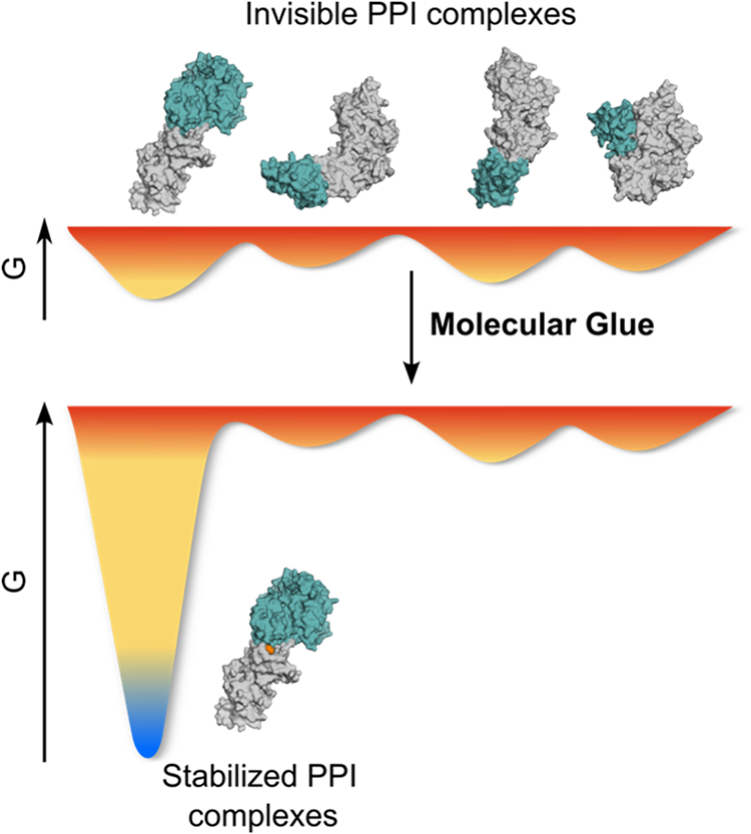
Conformational selection theory explains the mechanism of action of MGs. The presence of a molecular glue turns a short-lived binary complex into a long-lived ternary complex.

Proteins involved in a PPI often display complementary regions of hydrophobic residues in which a few of them, identified as hotspots, are critical for the affinity of the interaction. These hotspots are complemented by clusters of low-mobility residues known as stability patches that contribute to the protein–protein binding free energy. On the one hand, the low specificity of the hydrophobic effect explains why, if shape complementarity is maintained, a single molecular surface can interact with multiple partners. However, it is difficult to extract robust design principles for MGs solely on the basis of increasing the hydrophobic surface area. The O-ring theory, on the other hand, proposes that predesolvated polar interactions surrounded by hydrophobic residues play a critical role in stabilizing certain PPIs, even in the absence of large hydrophobic patches.^19,20^ Therefore, identifying these interactions and learning to use MGs to modulate their contribution to the overall stability may be the key to the prospective design of these molecules.

We recently demonstrated that in the lenalidomide-induced complex between Cereblon (CRBN) and Casein Kinase 1α (CK1α), the hydrophobic shielding effect triggered by the MG resulted in a significant increase in the strength of three key H-bond interactions at the protein–protein interface.^21^ Although the eventual protein degradation may depend on several contacts between the CRBN and the neosubstrates, the strength of the three key H-bonds was an excellent predictor of ternary complex stability. In that case, the binary complex of CRBN and CK1α would apparently be mediated through three polar interactions that sit within an incomplete O-ring, whose shape is complementary to the chemical structure of lenalidomide. In the ternary complex, the O-ring is completed and the polar interactions are much more robust. In this work, we explore the generalizability of the concept of incomplete O-rings, seeking to determine whether four chemically diverse molecular glues act via a common mechanism of action in their structurally unrelated protein partners. Specifically, we evaluate the effect of three different Immunomodulatory drugs (IMiDs) on the complex formed by CRBN and SALL4, a transcription factor essential for limb development. Next, we analyze the nanobody sensor of cannabidiol used by Cao et al. to redefine the concept of MGs. Third, we evaluated if Fusicoccin A (FC-A) can strengthen the electrostatic and polar interactions present in its ternary complex with 14-3-3σ and SSBP4. Finally, we study the effects of indisulam on the PPI between RBM39 and DCAF15. We describe how, although the strengthening of polar interactions is a reproducible phenomenon that extends beyond ternary complexes involving CRBN, the molecular determinants of PPI stabilization are as complex as those of the formation of PPIs itself. Based on these results, we propose a new methodological framework to evaluate MGs and contribute to their prospective design.

## Methods

### Molecular Simulations Setup

The models for the ternary complexes were built using the Protein Data Bank (PDB)^22–24^ crystallographic structures with PDB IDs 7BQU^25^ (CRBN–SALL4), 7TE8 ^12^ (cannabidiol nanosensor), 7OBX^26^ (14-3-3σ–SSBP4 structure), 7OBY^26^ (14-3-3σ–SSBP4 ternary complex with FC-A), and 6UD7 ^27^ (DCAF15–RBM39). Standard protein preparation protocols were followed, including the extraction of the biological assembly and the removal of crystallization buffer compounds and salts. Moreover, DNA damage-binding protein 1 (DDB1) and DET1- and DDB1-associated protein 1 (DDA1) were omitted in the DCAF15–RBM39 system. The appropriate capping groups were added to the terminal residues. The ff14SB^28^ and gaff2 ^29^ force fields were used to assign atom types for the protein and ligands, respectively. Parameters for the phosphorylated serine of SSBP4 were retrieved from the AMBER Parameter Database.^30^ Partial charges for the ligands were derived by using the AM1-BCC charging scheme. The Zn^2+^ cations bound to CRBN and SALL4 were parameterized using the bonded model utilizing ZAFF.^31^ Each system was solvated on a truncated octahedral box of TIP3P^32,33^ water molecules, and the appropriate quantities of counterions were added to achieve charge neutrality. Each system was then minimized in two stages. First, the position of water molecules was minimized, combining 3500 steps of steepest descent and 6500 steps of conjugate gradient, while the position of the proteins and ligand atoms was restrained using a harmonic potential with a force constant of 5.0 kcal/mol·Å^2^. Next, the protein and water molecules were minimized using 4500 steps of steepest descent, followed by 7500 steps of conjugate gradient, while the atoms of the molecular glues were restrained with a harmonic potential using the same force constant. The systems were then heated in the NVT ensemble from 100 to 298 K in three stages of 250 ps (100–150 K, 150–250 K, and 250–298 K). Subsequently, their density was equilibrated to 1 bar for 2 ns in the NPT ensemble. During the equilibration and subsequent production molecular dynamics (MD) trajectories, temperature control was achieved using a Langevin thermostat (with a collision frequency of 3 ps^–1^), and a Monte Carlo barostat was used to control the pressure when simulated in the NPT ensemble. SHAKE^34^ was applied to all atoms involving hydrogen to allow for a time step of 2 fs, and all simulations were performed with the CUDA accelerated version of PMEMD.^35^

### Steered Molecular Dynamics Protocol

The stability of the hydrogen bonds was assessed by performing 25 independent canonical ensemble Steered Molecular Dynamics (SMD) trajectories for each measured interaction conducted in three stages. First, new velocities were assigned to the equilibrated structure using a different random seed number at 298 K and an MD trajectory was performed for 10 ns, using flat-bottom restraints to keep all the protein–protein H-bonds at the interface between 2.5 and 3.5 Å using a force constant of 60 kcal/mol·Å^2^. Second, the final frame of this trajectory was used as a starting structure for a short 1 ns SMD simulation in which the donor and acceptor involved in the H-bond to be measured were pulled to closer proximity. Finally, an SMD simulation of 5 ns was started, in which the distance between donor and acceptor was increased at a rate of 0.5 Å/ns, using a spring constant of 500 kcal/mol·Å^2^ to ensure the applicability of the stiff spring approximation.^36^ Some of the measured interactions (Figure S1) involved rotatable and symmetric functional groups (e.g., carboxylic acids from D and E side chains) in which increasing the distance to one of these atoms was not enough to strain the interaction. In those cases, the distance to be steered was defined with respect to the center of mass of the symmetric group, and the distance thresholds were adjusted by 0.5 Å. In the case of interactions involving phosphorylated amino acids (i.e., 14-3-3σ–SSBP4), the protocol required further modification to account for the higher order of symmetry of the fully deprotonated phosphate group. Specifically, we leveraged the number-of-bonds collective variable, as implemented in the AMBER22 suite (eq 1),

1 $$\mathrm{CV}=\sum_{p}\frac{1-{\left(r_{p}/r_{0}\right)}^{6}}{1-{\left(r_{p}/r_{0}\right)}^{12}}$$

where the sum runs over pairs of atoms *p*, *r_p_* denotes the distance between the atoms of pair *p*, and *r*_0_ is a parameter measured in Å. The PMF_HB_break_ was computed using Jarzynski’s equality^37,38^ (eq 2) over the set of trajectories for each interaction,

2 $$e^{-\Delta G/k_{B}T}=\left\langle e^{-W_{i}/k_{B}T}\right\rangle$$

where the right-hand term corresponds to the ensemble average of exponential work values obtained in nonequilibrium conditions. From the above equation, for every step of the collective variable, the Potential of Mean Force (PMF) was obtained using eq 3,

3 $$\mathrm{PM}F_{\mathrm{HB}\_\mathrm{break}}=-k_{B}T\ln\sum_{i=1}^{N}\frac{e^{W_{i}^{\mathrm{HB}\_\mathrm{break}}/k_{B}T}}{N}$$

where *W_i_* ^HB_break^ refers to the work value of the *i*th independent SMD trajectory and *N* is the number of independent SMD trajectories (*N* = 25 in this work). The reported PMF_HB_break_ was calculated by applying eq 3 to the maximum work after the minimum value in each of the SMD replicates. To evaluate the statistical significance of the PMF_HB_break_ differences between the binary and ternary complex systems, we have performed a Mann–Whitney *U* nonparametric test^39^ on the work distributions. Error estimations for PMF were obtained by bootstrapping 50 times at each distance point of the set of work values. The total calculated PMF value for the complex corresponds to the sum of PMF_HB_break_ for the individual interactions that presented statistically significant differences and were uncorrelated.

### Calculation of Water Radial Distribution Function

The radial distribution function (RDF, g­(*r*)) is the ratio between the average number of water molecules found around certain atoms at a distance (*r*) and the bulk solvent along an MD simulation. The radial distribution function (RDF) of water molecules around the atoms involved in each interaction was obtained using CPPTRAJ.^40,41^ The RDF was calculated from the first step of the non-steered initialization stage, in which restraints were applied to the atoms involved in the interactions of interest. The calculation considered the range between 0 and 10 Å from the atoms of interest and used a bin spacing value of 0.1 Å. The water grids were calculated using the Grid Inhomogeneous Solvation Theory (GIST) approach as implemented in CPPTRAJ.^42,43^

## Results and Discussion

### IMiDs Recruit CRBN Neosubstrates through a Common Mechanism of Action

IMiDs such as thalidomide and its derivatives lenalidomide and pomalidomide are perhaps the landmark examples of therapeutic molecular glues. They bind to the E3 ubiquitin ligase CRBN and promote the recruitment of proteins that are not natural substrates, such as the transcription factors Ikaros (IKZF1) and Aiolos (IKZF3) and the enzyme CK1α, leading to their ubiquitination and subsequent degradation. These drugs exemplify the potential of MGs to impact human health, as they are widely used in the clinic to treat multiple hematologic malignancies. Therefore, we first evaluated the effect of pomalidomide (POM), thalidomide (THA), and lenalidomide (LEN) on the CRBN–SALL4 complex to investigate if the H-bond strengthening effect induced by lenalidomide on the CRBN–CK1α system is a general mechanism for other IMiDs and CRBN neosubstrates. The second zinc finger domain of SALL4 (SALL4^ZF2^) presents the conserved C2H2-ZF motif (C-X-X-C-G) identified as the IMiD-induced CRBN degron. The CRBN–SALL4^ZF2^ interaction is mediated by three key H-bonds: the side chains of CRBN residues N351, H357, and W400 interact with the backbone carbonyl oxygens of the SALL4^ZF2^ residues S413, V414, and C415. Moreover, the IMiDs establish three H-bonds with residues N351, H378, and W380 of CRBN but no polar interactions with SALL4. Using SMD simulations to measure the work required to separate the atoms involved in the H-bond from 2.5 to 5.0 Å, we compared the potential of mean force (PMF_HB_break_) needed to break separately each of these protein–protein interactions, henceforth referred to as CRBN^N351^–SALL4^S413^, CRBN^H357^–SALL4^V414^, and CRBN^W400^–SALL4^C415^ in the presence and absence of the IMiDs. The work profiles for each individual interaction were Boltzmann-averaged to obtain a potential of mean force profile along the breaking simulation, and the PMF_HB_break_ values were calculated by applying Jarzynski’s equality to the maximum work value beyond the equilibrium distance from each replica. In the absence of the molecular glues, all H-bonds presented low PMF_HB_break_ values, but it was especially noticeable in the case of CRBN^W400^–SALL4^C415^ in which the H-bond was very labile: 1.3 ± 0.2 kcal/mol compared to 3.2 ± 0.3 and 2.7 ± 0.3 kcal/mol in CRBN^N351^–SALL4^S413^ and CRBN^H357^–SALL4^V414^, respectively (Figure 2 and Table 1). In contrast, when any of the IMiDs was present, the PMF_HB_break_ values were significantly higher in all cases, with CRBN^N351^–SALL4^S413^ becoming the strongest interaction in the ternary complexes with the three IMiDs (PMF_HB_break_ ca. 9 kcal/mol). Interestingly, the effect of the three compounds varied across the different interactions, and pomalidomide and thalidomide seemed to display a higher stabilizing effect on the CRBN^H357^–SALL4^V414^ and CRBN^W400^–SALL4^C415^ interactions than lenalidomide (Figure 2 and Table 1).

**Figure 2:**
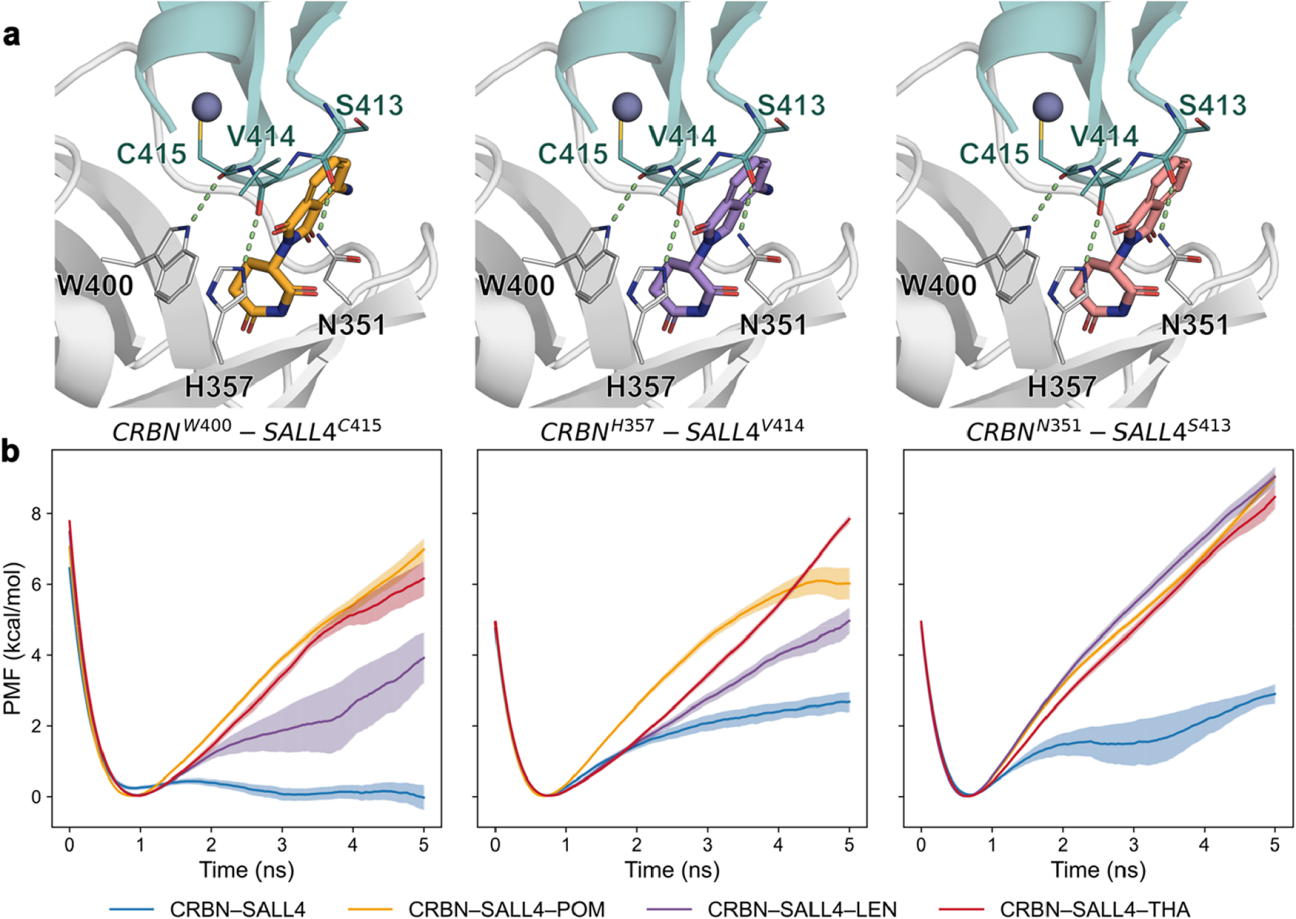
H-bond dissociation energy profiles in the presence and absence of IMiDs at the CRBN–SALL4 interface. (a) Detailed view of the CRBN (gray) and SALL4 (teal) interface in complex with pomalidomide (orange), lenalidomide (purple), or thalidomide (pink). (b) Energy profiles of the CRBN^W400^–SALL4^C415^ (left), CRBN^H357^–SALL4^V414^ (center), and CRBN^N351^–SALL4^S413^ (right) H-bonds in the absence and presence of pomalidomide, lenalidomide, and thalidomide. Starting computational models were built from the crystallographic structure with the PDB ID 7BQU.

**Table 1:** Summary of the PMF_HB_break_ Values in kcal/mol for the CRBN–SALL4 Systems Evaluated in This Work^a^

	CRBN–SALL4	CRBN–SALL4–POM	CRBN–SALL4–LEN	CRBN–SALL4–THA
CRBN^W400^–SALL4^C415^	1.3 ± 0.2	7.0 ± 0.3	3.9 ± 0.7	6.2 ± 0.7
		(+5.7 ± 0.4)	(+2.6 ± 0.7)	(+4.9 ± 0.7)
CRBN^H357^–SALL4^V414^	2.7 ± 0.3	6.3 ± 0.3	5.0 ± 0.5	7.8 ± 0.1
		(+3.6 ± 0.4)	(+2.3 ± 0.6)	(+5.1 ± 0.3)
CRBN^N351^–SALL4^S413^	3.2 ± 0.3	9.0 ± 0.1	9.0 ± 0.2	8.5 ± 0.3
		(+5.8 ± 0.3)	(+5.8 ± 0.4)	(+5.3 ± 0.4)
∑PMF_HB_break_	7.2 ± 0.5	22.3 ± 0.4	17.9 ± 0.9	22.5 ± 0.8
		(+15.1 ± 0.6)	(+10.7 ± 1.0)	(+15.3 ± 0.9)

a Relative values (in parentheses) are shown with respect to the CRBN–SALL4 binary complex. Error estimates were obtained by bootstrapping 50 times the work profiles used to estimate the PMF.

Although there is no experimental measurement that corresponds directly to the PMF_HB_break_ of a hydrogen bond, we had demonstrated previously that the stability of the ternary complex was correlated with the sum of PMF_HB_break_ for the three interactions, as long as the breaking of each of the H-bonds was uncorrelated with the distance of the remaining two (Figure S2 and Table S1). Indeed, the ternary complexes with each of the three IMiDs displayed a significant increase in the sum of the PMF_HB_break_ values with respect to the binary complex. Interestingly, the rank of this increase in the predicted strength of the protein–protein interaction is in qualitative agreement with the rank of the ternary complex stability as determined by TR-FRET.^44^ Specifically, the CRBN–SALL4^ZF2^ complex induced by pomalidomide and thalidomide displays similar *K*_D_ values (in line with the observed Δ∑PMF_HB_break_ = 15.3 ± 0.9 kcal/mol and Δ∑PMF_HB_break_ = 15.1 ± 0.6 kcal/mol predicted for thalidomide and pomalidomide, respectively), while the *K*_D_ for the complex induced by lenalidomide is slightly higher, in line with the slightly lower effect on the strength of the protein–protein interaction (Δ∑PMF_HB_break_ = 10.7 ± 1.0 kcal/mol). Solvent-exposed H-bonds are notoriously more labile than those buried in the core of the protein,^45,46^ as incoming water molecules can facilitate the separation of donor and acceptor. In line with our previous observations for the CRBN–CK1α–lenalidomide system, the presence of the molecular glue at the CRBN–SALL4^ZF2^ interface resulted in a significantly decreased accessibility of water molecules to the protein–protein interface, as reflected by the radial distribution function (RDF) of water molecules around the three H-bonds over the course of the MD trajectories (Figure S3). Interestingly, while the strengthening effect of the three IMiDs on CRBN^N351^–SALL4^S413^ and CRBN^H357^–SALL4^V414^ is aligned with their stabilization effect, it does not correspond to their ability to shield the hydrogen bonds at the interface when the complex is fully formed. This mismatch between the water accessibility to the equilibrium structure and strength of polar interactions replicates what we described previously for some mutations in CK1α and highlights that the strengthening of polar interactions stems from different factors that cannot be easily rationalized from static structure or equilibrium MD simulations. In contrast, our results demonstrate that measuring the combined strength of the hydrogen bonds offers a less ambiguous metric to estimate the overall stability of the ternary complex in CRBN-mediated systems, thus potentially providing crucial information for the prospective design of MGs targeting different neosubstrates of CRBN. Furthermore, this raised the question of whether this effect could be extended to other non-IMiD MGs beyond the CRBN-engaging chemical space.

### Ad Hoc Designed MG System Exhibits Stronger H-Bonds in the Ternary Complex

To determine whether the ability to strengthen protein–protein H-bonds is a mechanism of action common to other molecular glues, we first analyzed the dual-nanobody cannabidiol (CBD) sensor developed by Kang et al.^47^ to exemplify the canonical MG behavior. This biosensor works by chemically inducing the dimerization of two nanobodies: an anchor binder (CA14) and a matching dimerization partner (DB21), which heterodimerize only in the presence of CBD. The crystallographic structure of the ternary complex shows how cannabidiol is sandwiched between the two nanobodies, and all three components are making extensive intermolecular contacts. The resorcinol ring of cannabidiol is forming a single hydrogen bond with the side chain of D34 from CA14, and both nanobodies are forming a broad network of up to 17 H-bonds at the protein–protein interface (Figure 3A). We compared the work needed to break them in the presence and absence of cannabidiol. Eight of these interactions (CA14^R46^–DB21^F103^, CA14^R46^–DB21^L104^, CA14^S54^–DB21^E45:COM^, CA14^N55^–DB21^R46^, CA14^Y61^–DB21^D59^, CA14^D64:COM^–DB21^D59^, CA14^D64^–DB21^D59:COM^, and CA14^A108^–DB21^M106^) did not show statistically different work distributions (Mann–Whitney *U* *p*-value >0.05). In general, these interactions were established between flexible side chains, and their exposure to incoming water molecules was not affected by the presence of CBD. Furthermore, in contrast to the CRBN–SALL4^ZF2^ system, the contact network of the remaining interactions was more intricate, and some residues of the protein–protein interface simultaneously participated in more than one H-bond. We reviewed the correlation between the distances at the time of disrupting the different contacts and discarded highly correlated interactions (Pearson correlation coefficient >0.5) to avoid overestimating the ∑PMF_HB_break_. A summary of the correlation analysis among different interactions is provided in Figure S4 and Table S2. The remaining six interactions (Figure 3) displayed a significant difference in the distribution of works and were notably strengthened in the ternary complex with respect to the complex in the absence of CBD (Table 2), amounting to a Δ∑PMF_HB_break_ of 14.4 ± 1.3 kcal/mol. As in the case of the IMiDs, the presence of CBD hampered the access of water to the protein–protein interface, but once again, the magnitude of the strengthening effect was not directly predictable from the analysis of the RDF or the crystallographic structure (Figure S5). Taken together, these results suggest that stronger polar interactions may underlie the high cooperativity of this complex (α = 442) and highlight the value of assessing the strength of the protein–protein H-bonds beyond the analysis of static structures, either from X-ray crystallography or computational predictions obtained from machine learning models.

**Figure 3:**
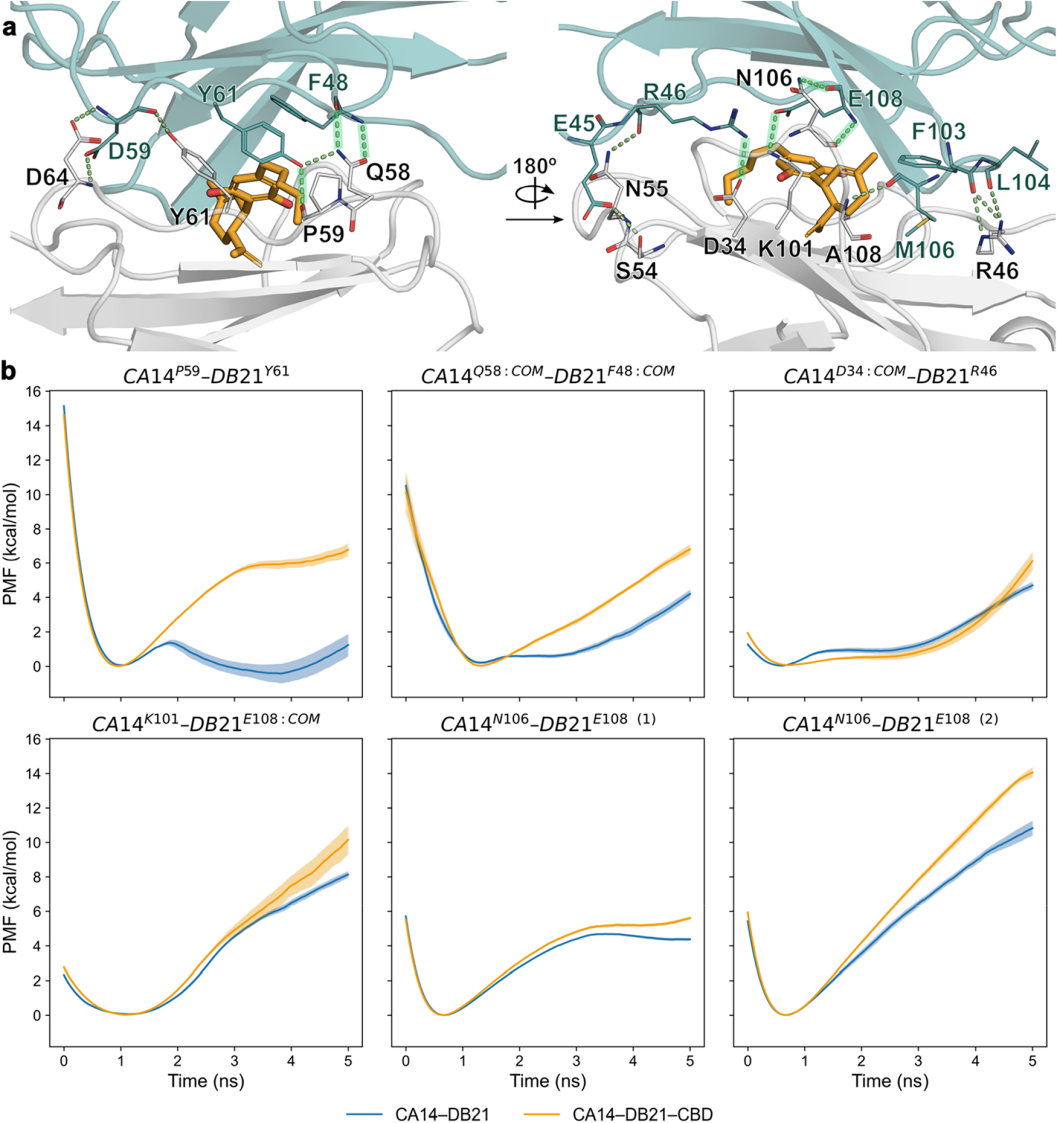
H-bond dissociation energy profiles in the presence and absence of cannabidiol at the CA14–DB21 interface. (a) Detailed view of the protein–protein interactions of the ternary complex formed by CA14 (gray), DB21 (teal), and cannabidiol (orange). The strengthened H-bonds are highlighted in green. (b) Energy profiles of the CA14^P59^–DB21^Y61^ (top left), CA14^Q58:COM^–DB21^F48:COM^ (top center), CA14^D34:COM^–DB21^R46^ (top right), CA14^K101^–DB21^E108:COM^ (bottom left), CA14^N106^–DB21^E108 (1)^ (bottom center), and CA14^N106^–DB21^E108 (2)^ (bottom right) interactions in the absence and presence of cannabidiol. The interaction CA14^N106^–DB21^E108 (1)^ corresponds to the H-bond between the side chain of N106 and the backbone of E108, and the interaction CA14^N106^–DB21^E108 (2)^ corresponds to the H-bond between the backbones of both residues. Starting computational models were built from the crystallographic structure with PDB ID 7TE8.

**Table 2:** Summary of PMF_HB_break_ Values (in kcal/mol) for the Cannabidiol Nanosensor Systems Evaluated in This Work^a^

	CA14–DB21	CA14–DB21–CBD
CA14^P59^–DB21^Y61^	2.6 ± 0.4	6.8 ± 0.3
		(+4.2 ± 0.5)
CA14^Q58:COM^–DB21^F48:COM^	4.2 ± 0.2	6.8 ± 0.3
		(+2.6 ± 0.4)
CA14^D34:COM^–DB21^R46^	4.7 ± 0.2	6.1 ± 0.5
		(+1.4 ± 0.5)
CA14^K101^–DB21^E108:COM^	8.1 ± 0.2	10.2 ± 0.8
		(+2.1 ± 0.8)
CA14^N106^–DB21^E108 (1)^	4.8 ± 0.1	5.6 ± 0.1
		(+0.8 ± 0.1)
CA14^N106^–DB21^E108 (2)^	10.8 ± 0.4	14.1 ± 0.3
		(+3.3 ± 0.5)
∑PMF_HB_break_	35.2 ± 0.7	49.6 ± 1.1
		(+14.4 ± 1.3)

a Relative values (in parentheses) are shown with respect to the CA14–DB21 binary complex. Error estimates were obtained by bootstrapping 50 times the work profiles used to estimate the PMF.

### Presence of Fusicoccin A Results in a Much More Robust Interaction of the Phosphorylated Residue in the 14-3-3σ–SSBP4 Complex

Fusicoccanes like Fusicoccin A (FC-A) or Cotylenin-A are natural products that stabilize the interactions between 14-3-3 proteins and their client proteins, influencing signaling pathways involved in cell survival and stress responses. Although the chemical complexity of fusicoccanes makes them unsuitable for further clinical advancement, they provide a good framework to study the underlying principles of the MGs mode of action. Previous studies revealed that FC-A induces a conformational change in 14-3-3σ to a “closed” conformation that accommodates the binding of the partner protein.^48,49^ However, we wanted to evaluate whether the presence of FC-A also resulted in stronger polar interactions that could account for the well-documented stabilization effect of FC-A across complexes involving the 14-3-3 family of proteins. To provide the proof of concept, we investigated the ternary complex of 14-3-3σ with single-stranded DNA-binding protein 4 (SSBP4) stabilized by FC-A.^26^ Similar to the CA14–DB21–CBD complex, the crystallographic structures of 14-3-3σ and the terminal region of SSBP4, with and without FC-A, show that there is an intricate network of 12 polar protein–protein interactions (Figure 4), including the interaction of the phosphorylated S384 residue in SSBP4 with residues R56, R129, and Y130 of 14-3-3σ. Furthermore, FC-A forms two hydrogen bonds with 14-3-3σ, one with the side chain of K122 and the other with the side chain of N215. To account only for the potential effect of FC-A on the strength of the different H-bonds, we analyzed the work profiles of all of the identified hydrogen bonds between the ternary complex and the same conformation after removing FC-A (hereafter referred to as non-FC-A structure). All of the interactions showed statistically significant differences in their work distributions. Interactions 14-3-3σ^K122^–SSBP4^V385:COM^ and 14-3-3σ^W230^–SSBP4^T382^ were not considered in the calculation of ∑PMF_HB_break_ due to their high correlation with interactions 14-3-3σ^N175:COM^–SSBP4^V385:COM^ and 14-3-3σ^N226:COM^–SSBP4^M383:COM,^ respectively (Figure S6 and Table S3). After these adjustments, the Δ∑PMF_HB_break_ between the ternary complex and the non-FC-A structure was calculated to be 11.7 ± 1.3 kcal/mol, (Table 3) with the main contribution coming from the breaking of the interactions between the phosphorylated S384 and residues R56, R129, and Y130 (6.6 ± 1.1 kcal/mol). The remaining interactions were calculated to contribute 2.4 ± 0.4 kcal/mol (14-3-3σ^N226:COM^–SSBP4^M383:COM^), 1.2 ± 0.3 kcal/mol (14-3-3σ^K49^–SSBP4^V385:COM^), and 1.5 ± 0.5 kcal/mol (14-3-3σ^N175:COM^–SSBP4^V385:COM^). Interestingly, when comparing the PMF profiles of the non-FC-A structure (PDB ID 7OBY) with the same polar interactions in the binary complex (PDB ID 7OBX), we found two interactions (14-3-3σ^N175:COM^–SSBP4^V385:COM^ and 14-3-3σ^K122^–SSBP4^V385:COM^) with statistically significant differences in their work distributions. However, the two interactions were strongly correlated (Pearson correlation coefficient >0.5), and their Δ∑PMF_HB_break_ values were less than 1 kcal/mol, resulting in a Δ∑PMF_HB_break_ of 0.6 ± 0.3 kcal/mol. Moreover, the interaction involving phosphorylated S384 did not display any significant difference (Figure S7). We hypothesize that the similar PMF profiles in this case could stem from 14-3-3σ spontaneously relaxing to a conformation similar to PDB ID 7OBX when FC-A is removed. In contrast to the previous cases, the analysis of the RDF profiles did not point to any significant increase in the hydrophobic shielding around the polar interactions in the ternary complex (Figures S8). However, the analysis of the Root Mean Square Fluctuation of the protein backbone revealed that α-helix 9 of 14-3-3σ is more flexible in the absence of FC-A (Figure S9), suggesting that, in the case of 14-3-3σ, the strengthening of polar interactions may arise from an increased rigidity of the overall complex, in line with what has been suggested previously in protein–ligand complexes.^50^

**Figure 4:**
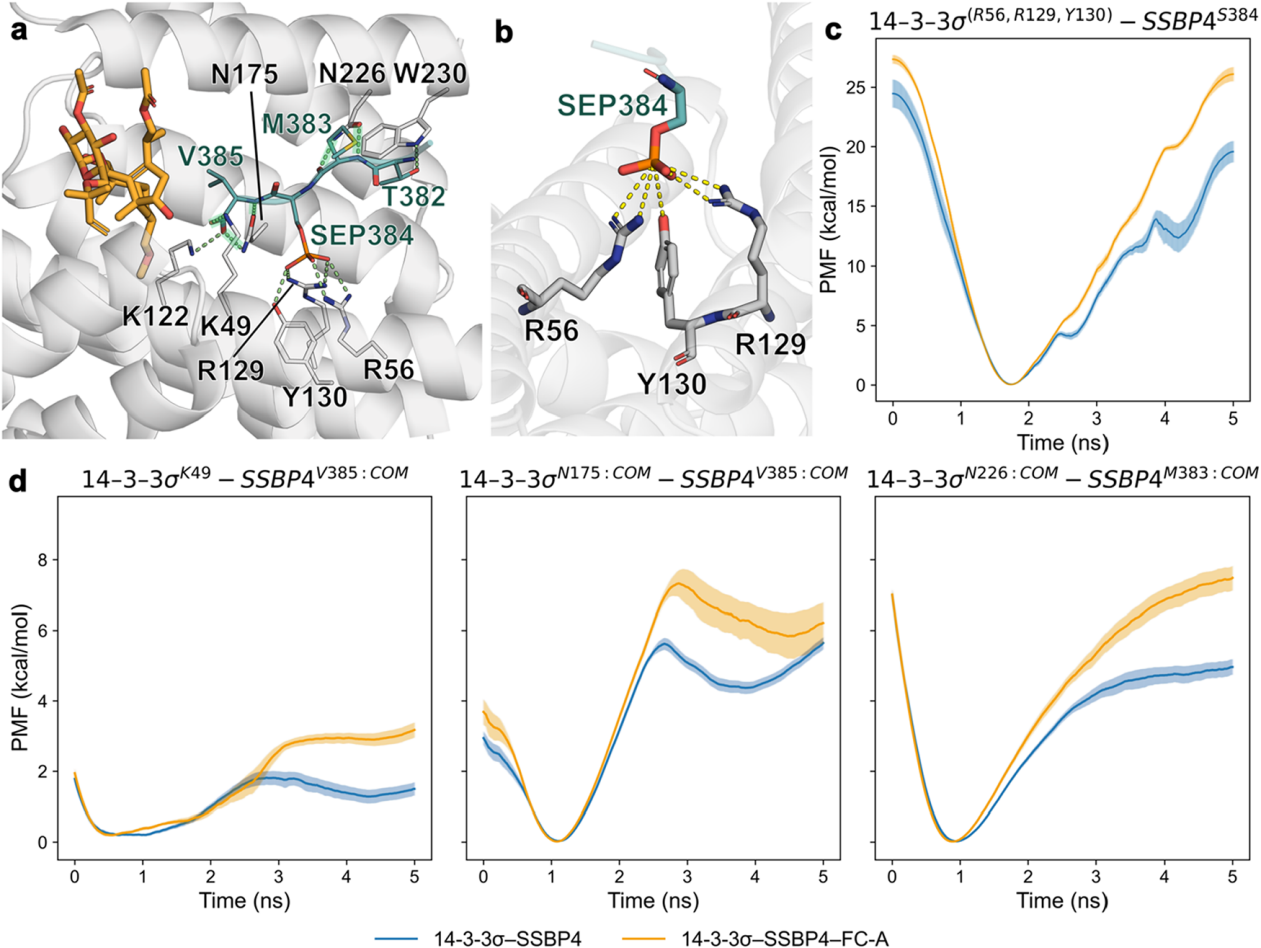
H-bond dissociation energy profiles in the presence and absence of FC-A at the 14-3-3σ–SSBP4 interface. (a) Detailed view of the interface between 14-3-3σ (gray) and the SSBP4 peptide (teal) in complex with FC-A (orange). The strengthened H-bonds are highlighted in green. (b) Detailed view of the distances included in the collective variable to measure the PMF_HB_break_ of the interactions with the phosphorylated S384 (c) Energy profiles of the 14-3-3σ^(R56,R129,Y130)^–SSBP4^S384^ interactions in absence and presence of FC-A. (d) Energy profiles of the 14-3-3σ^K49^–SSBP4^V385:COM^ (left), 14-3-3σ^N175^–SSBP4^V385^ (center), and 14-3-3σ^N226^–SSBP4^M383^ (right) interactions in the absence and presence of FC-A. Starting computational models were built from the crystallographic structure with PDB ID 7OBY.

**Table 3:** Summary of the PMF_HB_break_ Values (in kcal/mol) for the 14-3-3σ–SSBP4 Systems Evaluated in This Work^a^

	14-3-3σ–SSBP4	14-3-3σ–SSBP4–FC-A
14-3-3σ^(R56,R129,Y130)^–SSBP4^S384^	19.5 ± 0.9	26.1 ± 0.6
		(+6.6 ± 1.1)
14-3-3σ^K49^–SSBP4^V385:COM^	2.1 ± 0.2	3.3 ± 0.2
		(+1.2 ± 0.3)
14-3-3σ^N175:COM^–SSBP4^V385:COM^	6.2 ± 0.2	7.7 ± 0.5
		(+1.5 ± 0.5)
14-3-3σ^N226:COM^–SSBP4^M383:COM^	5.2 ± 0.2	7.6 ± 0.3
		(+2.4 ± 0.4)
∑PMF_HB_break_	33.0 ± 1.0	44.7 ± 0.9
		(+11.7 ± 1.3)

a Relative values (in parentheses) are shown with respect to the 14-3-3σ–SSBP4 non-FC-A complex. Error estimates were obtained by bootstrapping 50 times the work profiles used to estimate the PMF.

### Indisulam Induces a Network of New Interactions in the Ternary Complex

Indisulam and its aryl sulfonamide analogs are antitumoral compounds whose discovery and mechanism of action resemble those of IMiDs. First discovered in phenotypical assays, retrospective studies revealed that indisulam is a molecular glue that recruits RBM39 to DCAF15, the substrate receptor of the Rbx-Cul4-DDA1-DDB1-DCAF15 complex, leading to its ubiquitination and subsequent degradation by the proteasome. Indisulam presents certain affinity for DCAF15 (>50 μM) but no measurable binding to RBM39 alone.^27^ In the crystallographic structure of the ternary complex, it can be observed how the MG is bound to a pocket between RBM39 and DCAF15, establishing three H-bonds and one π-stacking interaction with DCAF15, plus a water-mediated H-bond with RBM39 (Figure 5b). Importantly, the central α-helix of RBM39 is placed in a cleft of DCAF15 establishing multiple nonpolar contacts and burying a large surface area, thus displaying higher chemical and shape complementarity than the other studied systems. There are ten additional peripheral protein–protein polar contacts, but many of them are solvent exposed and involve flexible residues (Figure 5a), both traits usually associated with weak interactions. Indeed, only the DCAF15^D557:COM^–RBM39^A317^ interaction presented statistically significant differences in the work distributions between the binary and the ternary complex, even though the ΔPMF_HB_break_ amounts to only −0.5 ± 0.2 kcal/mol (Figure 5c). These results suggest that the strengthening of protein–protein interactions is not the main driver of the gluing effect of indisulam. However, we investigated the strength of the hydrogen bonds established between the MG and its protein partners. The interactions established between indisulam and residues A234, F235, and F231 of DCAF15 are very weak in the absence of RBM39, but they are strengthened in the ternary complex (by 1.6 ± 0.5 and 2.5 ± 0.8 kcal/mol, respectively). In addition, the strength of the water-mediated interaction (4.3 ± 0.3 kcal/mol) is of a similar magnitude to the highest Δ∑PMF_HB_break_ measured in other systems, thus suggesting that it could provide a similar contribution to the overall stability of the ternary complex. Thus, in contrast to other MGs, indisulam acts as a linchpin in the protein–protein complex, becoming a crucial addition to an already highly complementary interface.

**Figure 5:**
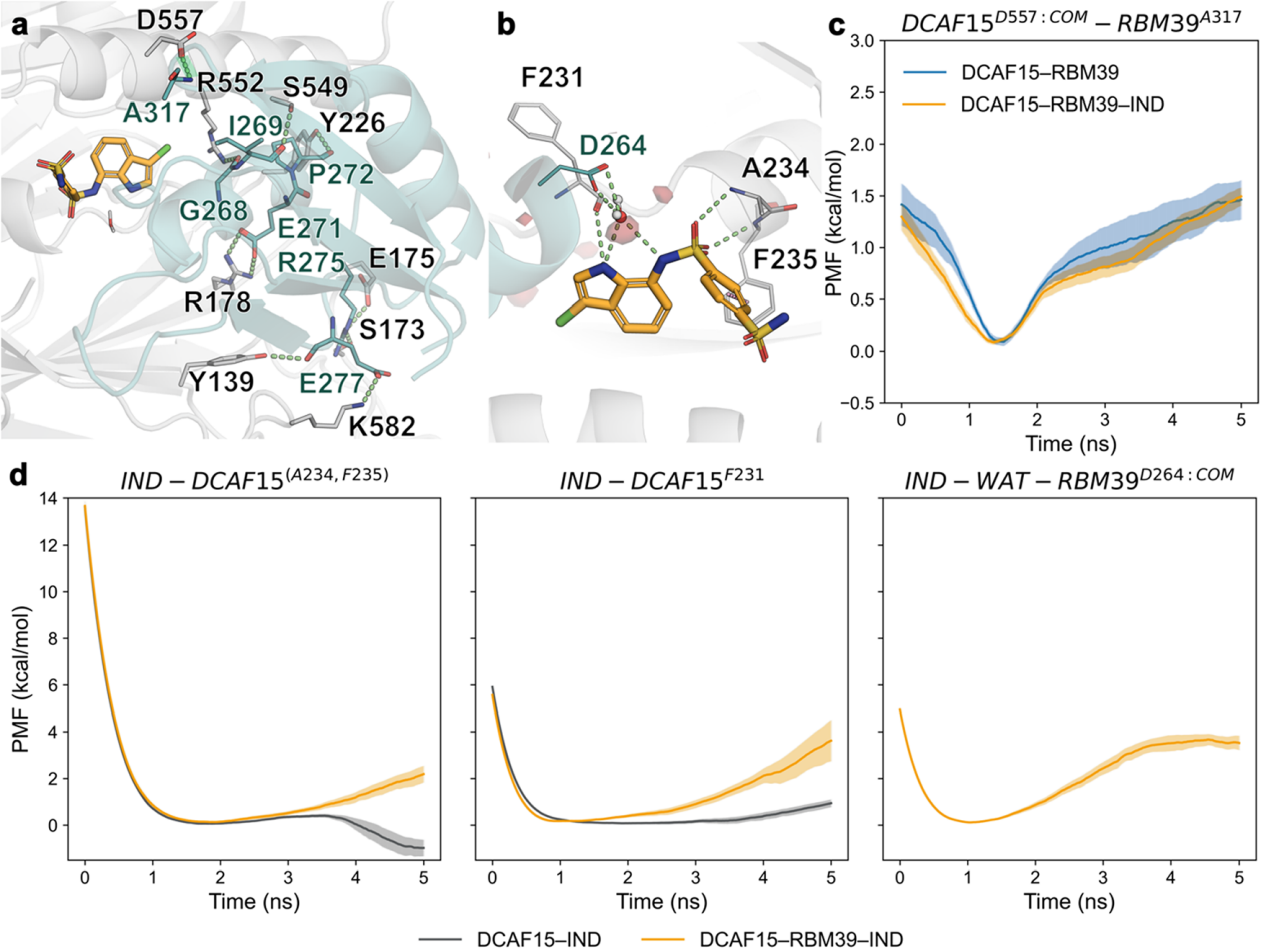
H-bond dissociation energy profiles in the indisulam-induced DCAF15–RBM39 complex. (a) Interface between DCAF15 (gray) and RBM39 (teal) in complex with indisulam (orange). The H-bond displaying statistically significant differences in the work distribution is highlighted in green. (b) Detailed view of the H-bonds formed between indisulam and DCAF15 and RBM39 during the molecular dynamics simulations. The water density at the binding site calculated in the sampling simulations is shown in red (isosurface at −4 kcal/mol/Å^3^). (c) Energy profiles of the DCAF15^D557:COM^–RBM39^A317^ interaction in the absence and presence of indisulam. (d) Energy profiles of the H-bonds formed between indisulam and the protein partners: IND–DCAF15^(A234,F235)^ (left), IND–DCAF15^F231^ (center), and IND–WAT–RBM39^D264:COM^ (right) interactions in the absence or presence of RBM39. Starting computational models were built from the crystallographic structure with PDB ID 6UD7.

### New Methodological Framework for the Prospective Design of MGs

In our view, the described results support that measures of affinity of ternary complexes and phenotypical approaches do not offer enough mechanistic insights to enable the use of structure-based drug design approaches in the prospective discovery of MGs. Instead, similar to the formation of PPIs, their stabilization is a complex phenomenon in which multiple contributions can play a role, and while one may dominate in specific cases, its identification *a priori* remains extremely challenging. For example, while a ligand-induced increase in the strength of polar interactions drives the behavior of three out of four of the analyzed MGs, the findings on the DCAF15–RBM39–indisulam system indicate that this is not a universal phenomenon. Our results also highlight that, at the structural level, MGs can achieve a similar outcome following different mechanisms. In the latter case, indisulam establishes a new network of interactions that seem to critically enhance the structural complementarity between the two protein partners. Based on our findings, we introduce a conceptual framework (Figure 6) in which each MG should be seen as occupying a niche in a spectrum depending on its structural role within the ternary complex. On one end of the spectrum, we would locate molecules that are able to further stabilize pre-existing PPIs, such as FC-A, which enhances the affinity between 14-3-3 proteins and phosphorylated peptides. Our results suggest that the gluing effect stems from a combination of a conformational change that occurs upon MG binding that rigidifies the complex^26^ and the strengthening of the polar interactions (including the interactions with the phosphate group). Therefore, accounting for both the rigidity of the complex and the strength of polar interactions will be necessary for the rational discovery of pharmacological modulators of this family of proteins, whose great therapeutic potential is yet to be realized. Further down the spectrum, there would be the dual-nanobody CBD sensor and the original IMiDs, which strengthen polar interactions by completing a pre-existing incomplete O-ring structure. In these cases, which would likely be characterized by protein contacts with low or non-observable experimental affinity, we propose a design strategy based on identifying those chemotypes that most enhance the robustness of polar contacts within the PPIs, as our results suggest that the overall strength of these contacts is a good predictor of stability of the ternary complex. In turn, the latest developments in PPI prediction^51,52^ may be leveraged for the computational identification of these incomplete O-rings. Importantly, expanding the therapeutic scope of IMiDs to target novel, clinically relevant neosubstrates is an extremely active research area in several pharmaceutical companies, with growing efforts to develop tools to enable the medicinal chemistry approaches for CRBN and to engage a broader range of targets.^53–59^ We put forward that evaluating the strength of the H-bonds at the protein–protein interface could be incorporated alongside other computational techniques to aid the discovery of novel MGs that engage CRBN. Notably, more recent IMiDs, such as CC-885 may be placed along the spectrum, as structural evidence suggests that these molecules also establish new interactions with their intended neosubstrate.^60^ Continuing down the spectrum, we can locate indisulam and similar molecules that are able to enhance a protein–protein interface by introducing new interactions but do not affect the strength of pre-existing polar interactions. Finally, on the opposite end of the spectrum from FC-A, we would find bifunctional molecules that do not rely on complementarity between protein partners and establish well-differentiated sets of intermolecular interactions with them.^61^ Because different types of MGs will require a different optimization strategy, positioning any new putative MG system along this spectrum will help choose the correct approximation (whether reinforcing already existing interactions at the PPI or attempting to generate new ones), thus aiding the discovery process of these molecules.

**Figure 6:**
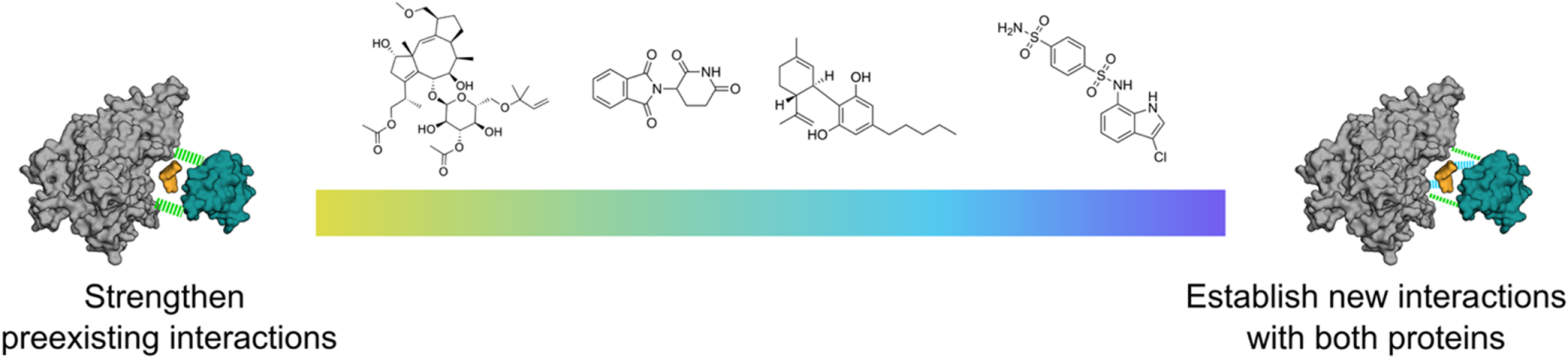
Schematic view of the conceptual spectrum of molecular glues, depending on their structural role within the ternary complex.

## Conclusions

By analyzing four canonical MG ternary complex systems, we have demonstrated that these molecules exhibit several system-dependent structural and dynamical intricacies that must be accounted for when pursuing their rational design. This complexity is further compounded by the chemical diversity of MGs and the significant impact that chemical modifications can have on the overall ternary complex. The combination of these factors makes deriving clear structure–activity relationships extremely challenging and underscores the need for innovative approaches beyond the traditional combination of computer-aided drug design and medicinal chemistry, which is designed to optimize affinity for a single target.

Our findings suggest that estimating the strengthening of polar interactions can serve as a useful predictor of increased stability of ternary complexes. In this context, we demonstrated that steered molecular dynamics is a very powerful tool for evaluating polar interactions, and it could be easily incorporated into MGs discovery workflows. Nevertheless, we have also demonstrated that the effect is not universal and arises from various contributing factors. Therefore, it is essential to carefully evaluate the driving forces behind each MG prior to pursuing optimization efforts. To this end, we propose shifting from a purely functional classification of MGs to a conceptual framework that categorizes them by the extent to which their mechanism of action relies on pre-existing protein–protein interactions or on generating new networks of molecular contacts. We anticipate that correctly positioning any putative molecular glue-induced system along this spectrum will aid in making informed design decisions, streamline the development process, and ultimately accelerate the therapeutic applications of these promising molecules.

## Supplementary Material



## Data Availability

The data sets supporting the findings of this study, including input files for each studied system, output work profiles, and analysis scripts, are publicly available in the Bitbucket repository at the following address: https://bitbucket.org/jjuarez84/mg_hbonds/src Additional data, including figures and tables, are provided in the Supporting Information (SI) accompanying this article.

## Notes

SMD setup for symmetric interactions (Figure S1); plots of distance along SMD simulations and average Pearson correlation coefficients between interactions used to assess the correlation among interactions at the interfaces of CRBN–SALL4 (Figure S2, Table S1); C14–DB21 (Figure S4, Table S2); and 14-3-3σ–SSBP4 (Figure S6, Table S3); RDF plots for polar interactions in CRBN–SALL4 (Figure S3); C14–DB21 (Figure S5); 14-3-3σ–SSBP4 (Figure S8); comparison of H-bond dissociation energy profiles for selected interactions in the absence of FC-A between the non-FC-A structure and the complex between 14-3-3σ–SSBP4 in the PDB ID 7OBX (Figure S7); change in the flexibility of the 14-3-3σ–SSBP4 triggered by the presence of FC-A (Figure S9); and description of the available input files and data sets and address of the repository (PDF)
