# Supplementary material for: Understanding the Role of H‑Bonds in the Stability of Molecular Glue-Induced Ternary Complexes
Source: J Chem Inf Model. 2026 Jan 23;66(6):3237–48. doi: 10.1021/acs.jcim.5c02718 (PMC13014453; doi:10.1021/acs.jcim.5c02718)
Supplement: Supplementary file 1 [file ci5c02718_si_001.pdf]

Supporting Information

Understanding the role of H-bonds in the stability of molecular glue-induced ternary complexes

Patricia Blanco-Gabella[1,2,3], Varbina Ivanova[1,2,3], Álvaro Serrano-Morrás[1,4], Julian E. Fuchs[5], Jordi Juárez-Jiménez[1,2]

[1] *Departament de Farmàcia i Tecnologia Farmacèutica, i Fisicoquímica, Facultat de Farmàcia i Ciències de l'Alimentació, University of Barcelona, Joan XXIII 27-31 08028, Barcelona, Espanya*

[2] *Institut de Química Teòrica i Computacional (IQTC), Facultat de Química i Física, Universitat de Barcelona (UB), C. Martí i Franqués, 1, 08028, Barcelona, Spain*

[3] *Institut de Biomedicina (IBUB), Facultat de Biologia, Universitat de Barcelona, Av. Diagonal 643, 08028, Barcelona, Espanya*

[4] *Boehringer Ingelheim RCV GmbH & Co. KG, Dr. Boehringer Gasse 5-11, 1121 Vienna, Austria*

* Corresponding author email: jordi.juarez@ub.edu

Table of Contents

Supplementary figures

Figure S1 S3
Figure S2 S4
Figure S3 S5
Figure S4 S6
Figure S5 S7
Figure S6 S8
Figure S7 S9
Figure S8 S10
Figure S9 S11

Supplementary tables

Table S1 S12
Table S2 S13
Table S3 S14

Available Datasets S15

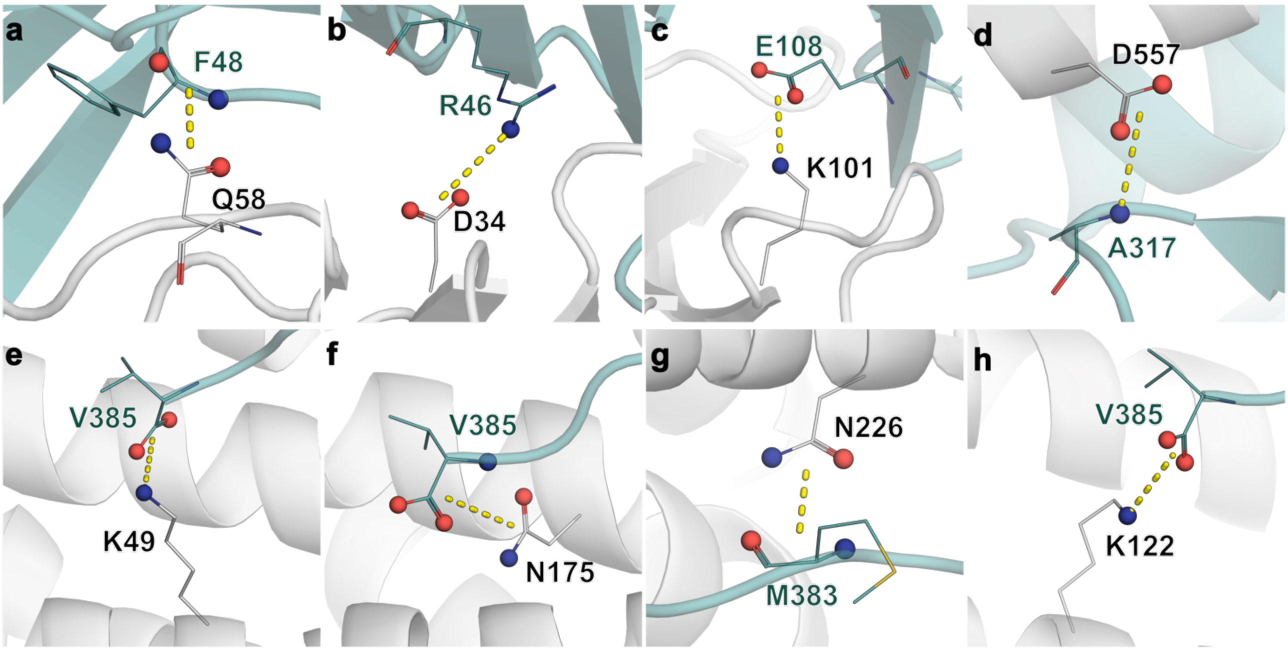

Figure S1. Atoms considered in the interactions measured using center of masses (COM). *The atoms used to calculate the work of the a) CA14$^{Q58:COM}$–DB21$^{F48:COM}$, b) CA14$^{D34:COM}$–DB21^{R46}, c) CA14^{K101}–DB21$^{E108:COM}$, d) DCAF15$^{D557:COM}$–RBM39^{A317}, e) 14-3-3σ^{K49}–SSBP4$^{V385:COM}$, f) 14-3-3σ$^{N175:COM}$–SSBP4$^{V385:COM}$, g) 14-3-3σ$^{N226:COM}$–SSBP4$^{M383:COM}$ and h) 14-3-3σ^{K122}–SSBP4$^{V385:COM}$ interactions are represented with spheres.*

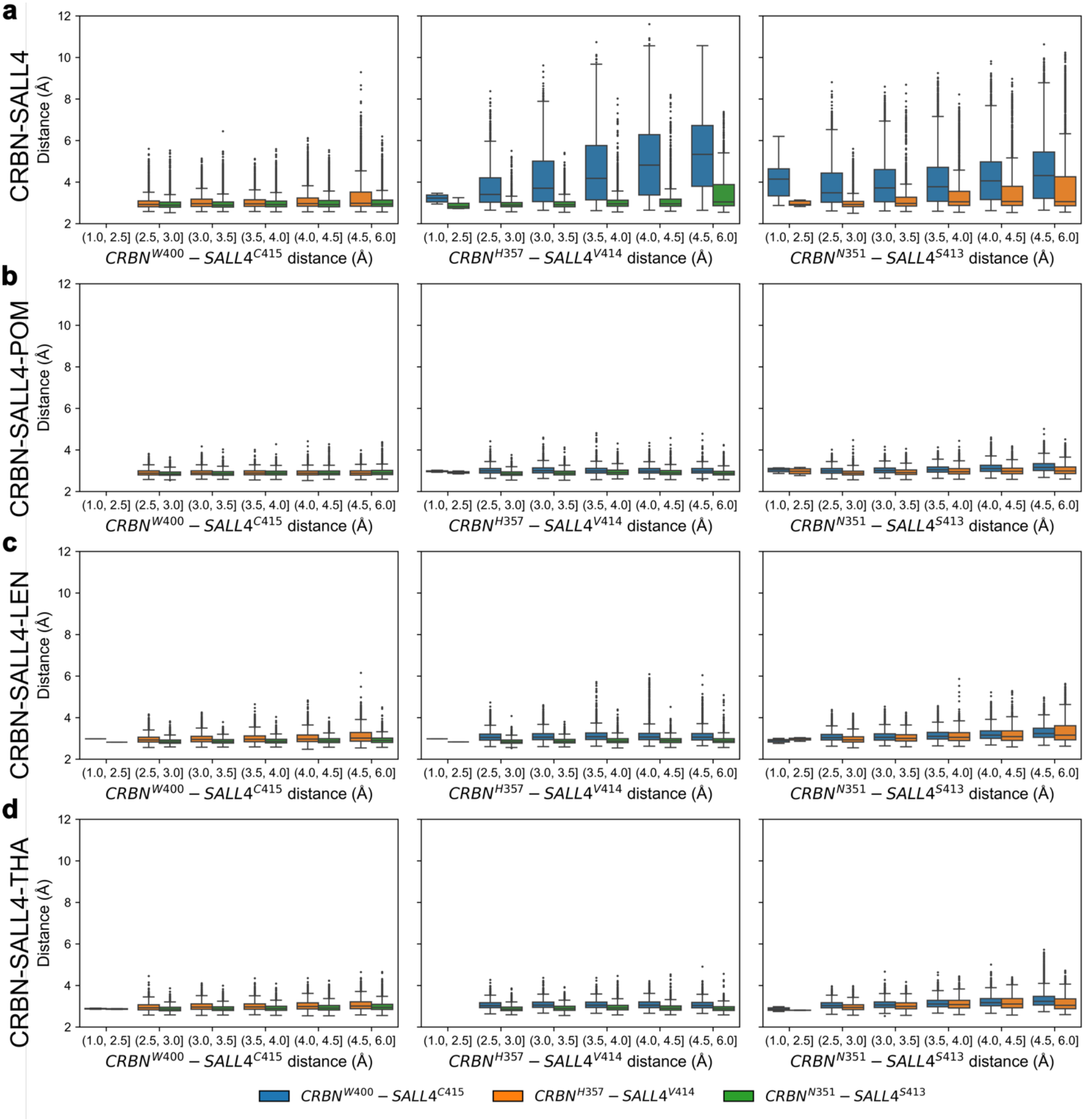

Figure S2. Distribution of distance values of the two H-bonds at the CRBN–SALL4 interface when the third is pulled apart in SMD trajectories. *a) Distribution of distance values in the apo trajectories. b) Distribution of distance values in the trajectories in complex with pomalidomide. c) Distribution of distance values in the trajectories in complex with lenalidomide. d) Distribution of distance values in the trajectories in complex with thalidomide. Average Pearson correlation coefficients (obtained from the inverse of the average Fisher's transformed correlation coefficients from individual trajectories) are reported in Table S1.*

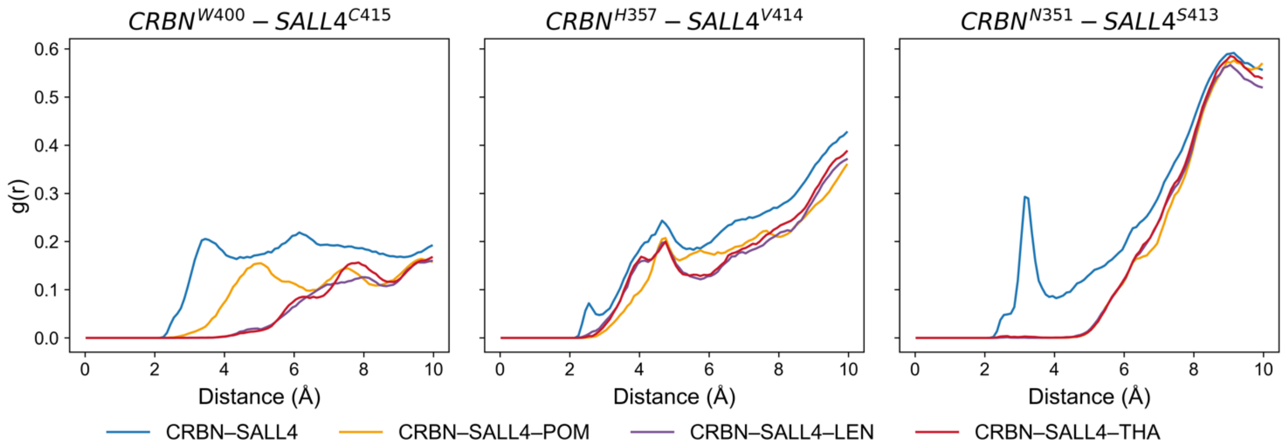

Figure S3. Water shielding effect of IMiDs in the CRBN–SALL4 system*. Radial distribution function of water around the atoms involved in the interactions considered. The interaction $CRBN^{W400}$–$SALL4^{C415}$ is on the left panel, $CRBN^{W400}$–$SALL4^{C415}$ is in the middle and $CRBN^{W400}$–$SALL4^{C415}$ is on the right panel. The binary complex is depicted in marine, the ternary complex with pomalidomide is depicted in orange, the ternary complex with lenalidomide is depicted in purple and the ternary complex with thalidomide is depicted in red.*

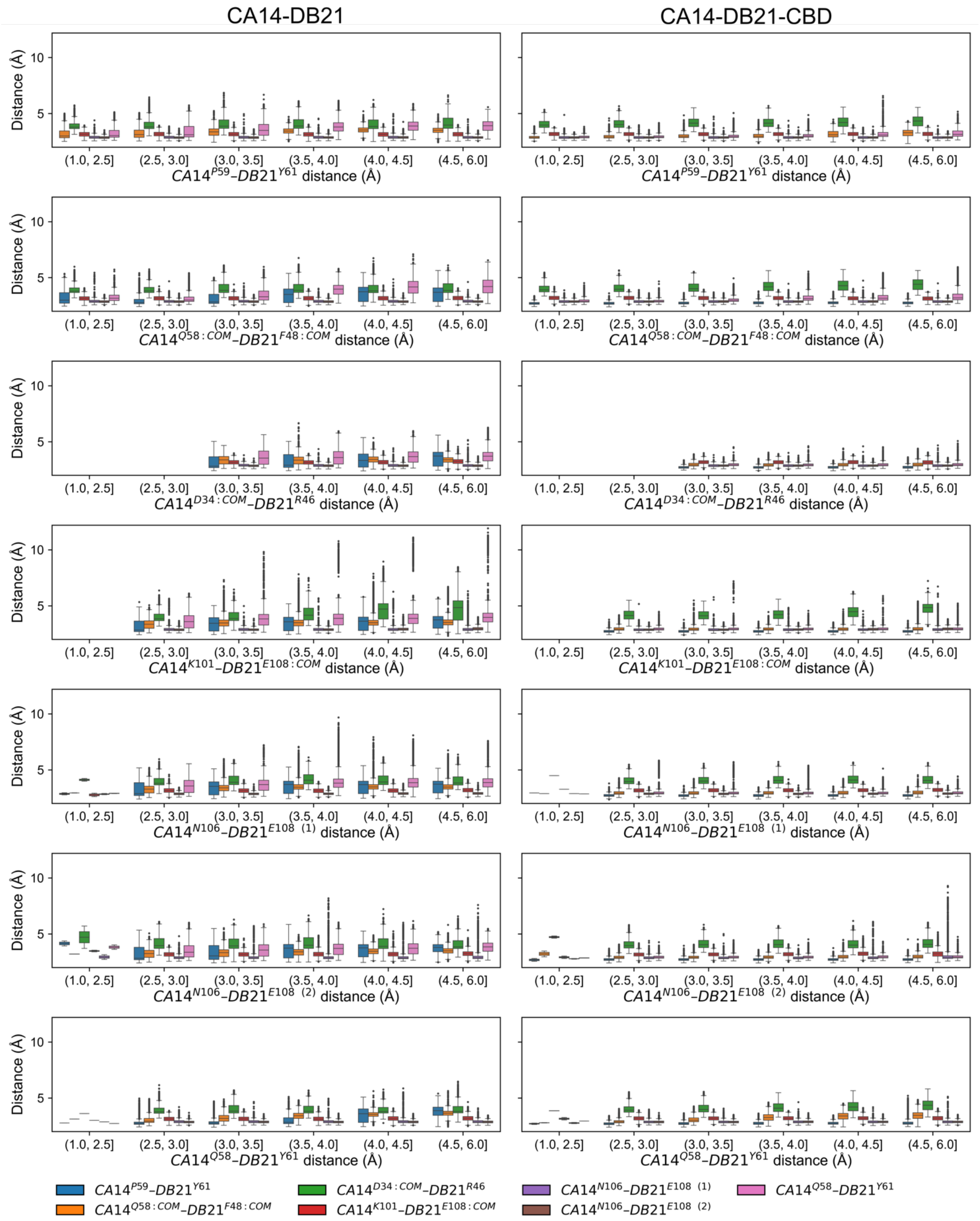

Figure S4. Distribution of distance values of the six interactions at the CA14–DB21 interface when the seventh interaction is pulled apart in SMD trajectories. *Left panel are the trajectories without cannabidiol, and right panel are the trajectories with cannabidiol. Average Pearson correlation coefficients (obtained from the inverse of the average Fisher's transformed correlation coefficients from individual trajectories) are reported in Table S2.*

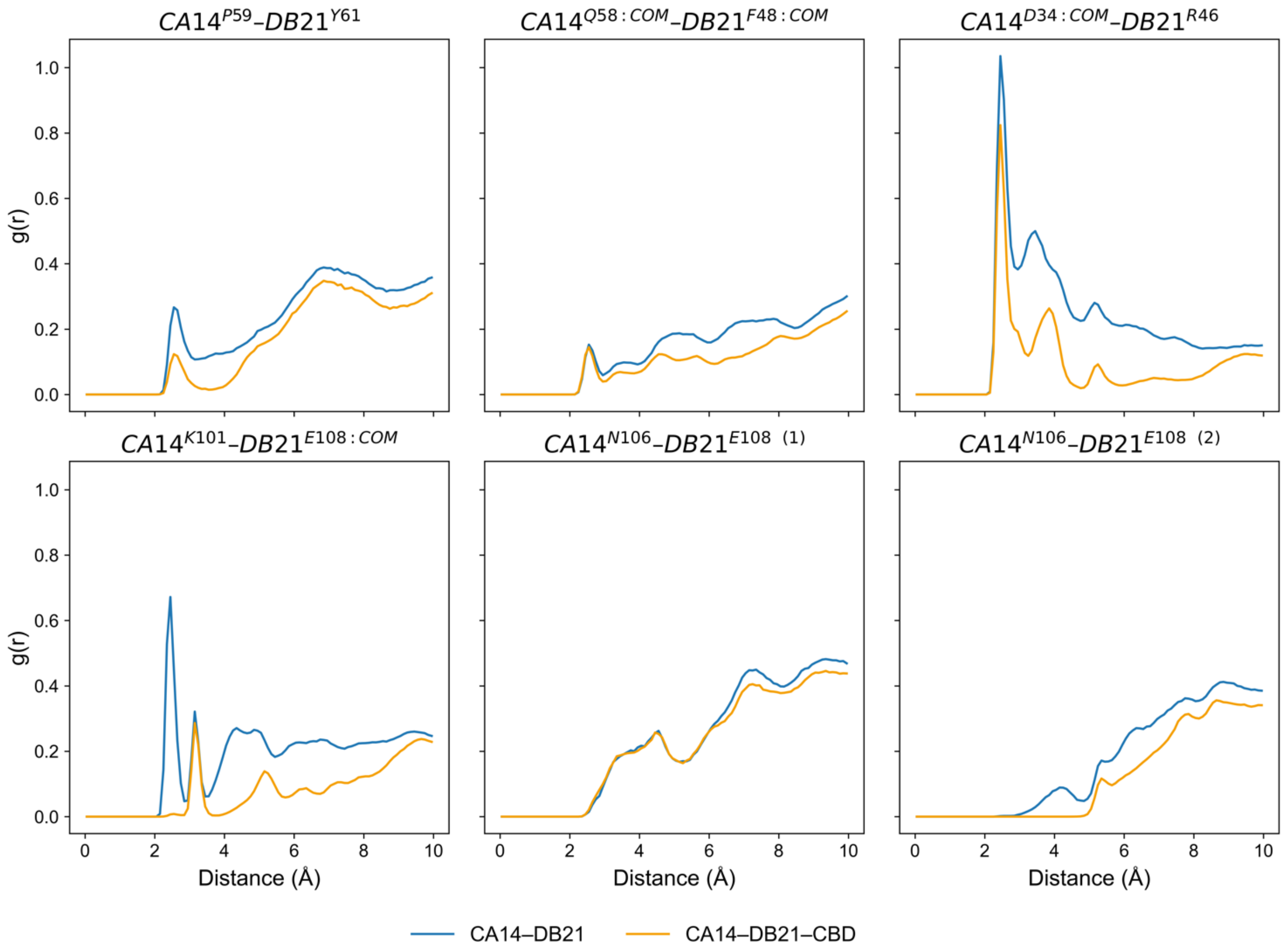

***Figure S5. Water shielding effect of CBD in the interactions considered in the cannabidiol nanosensor system**. Radial distribution function of water around the atoms involved in the interactions considered. The binary complex is depicted in marine and the ternary complex with cannabidiol is depicted in orange.*

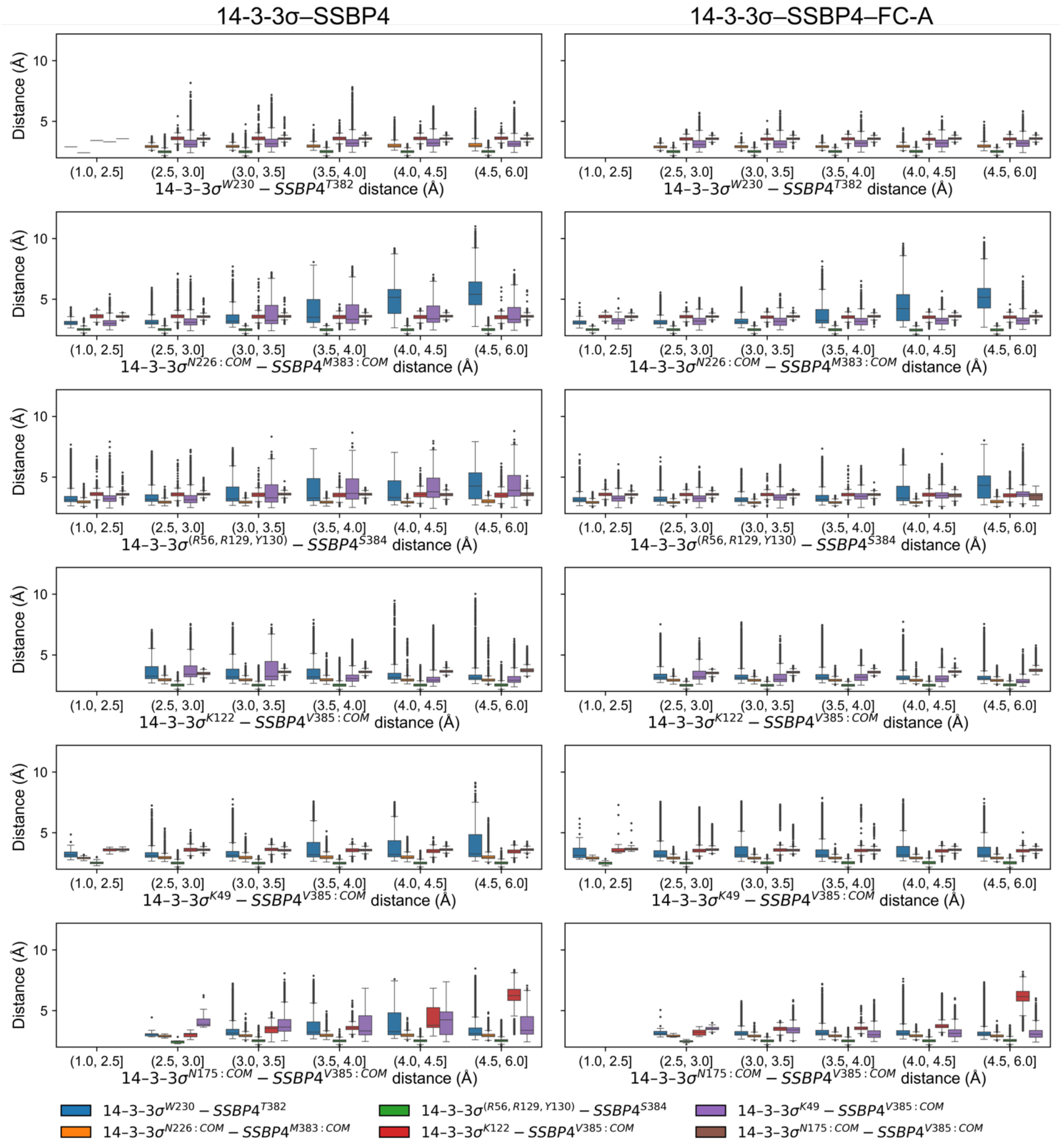

Figure S6. Distribution of distance values of the five interactions at the 14-3-3σ–SSBP4 interface when the sixth interaction is pulled apart in SMD trajectories. *Left panel are the trajectories without Fusicoccin A, and right panel are the trajectories with Fusicoccin A. Average Pearson correlation coefficients (obtained from the inverse of the average Fisher's transformed correlation coefficients from individual trajectories) are reported in Table S3.*

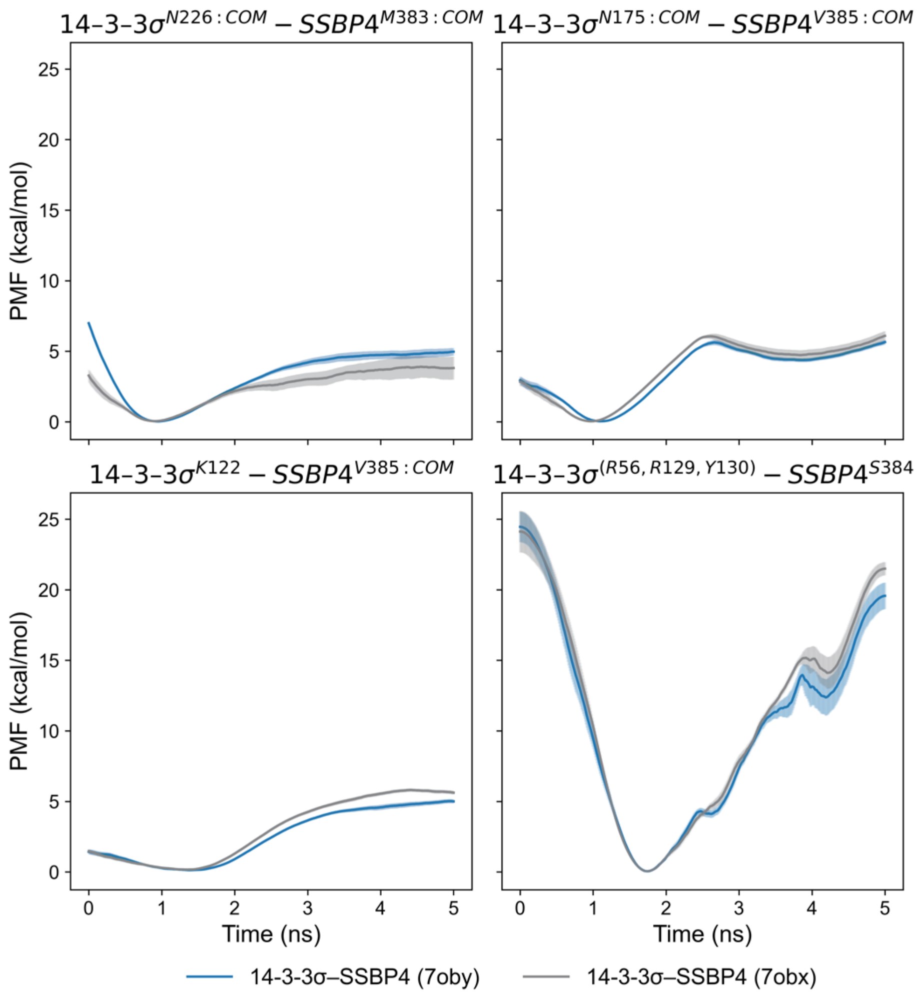

Figure S7. H-bond dissociation energy profiles of the 14-3-3σ–SSBP4 complex in absence of FC-A. *Energy profiles of the 14-3-3σ^{K122}–SSBP4$^{V385:COM}$ (left top), 14-3-3σ^{N175}–SSBP4^{V385} (left bottom) and 14-3-3σ^{N226}–SSBP4^{M383} (right top) and 14-3-3σ$^{(R56,\ R129,\ Y130)}$–SSBP4^{S384} (right bottom) interactions in the no-FC-A (PDB ID 7OBY) structure (in blue) and the binary complex (PDB ID 7OBX) structure (in gray).*

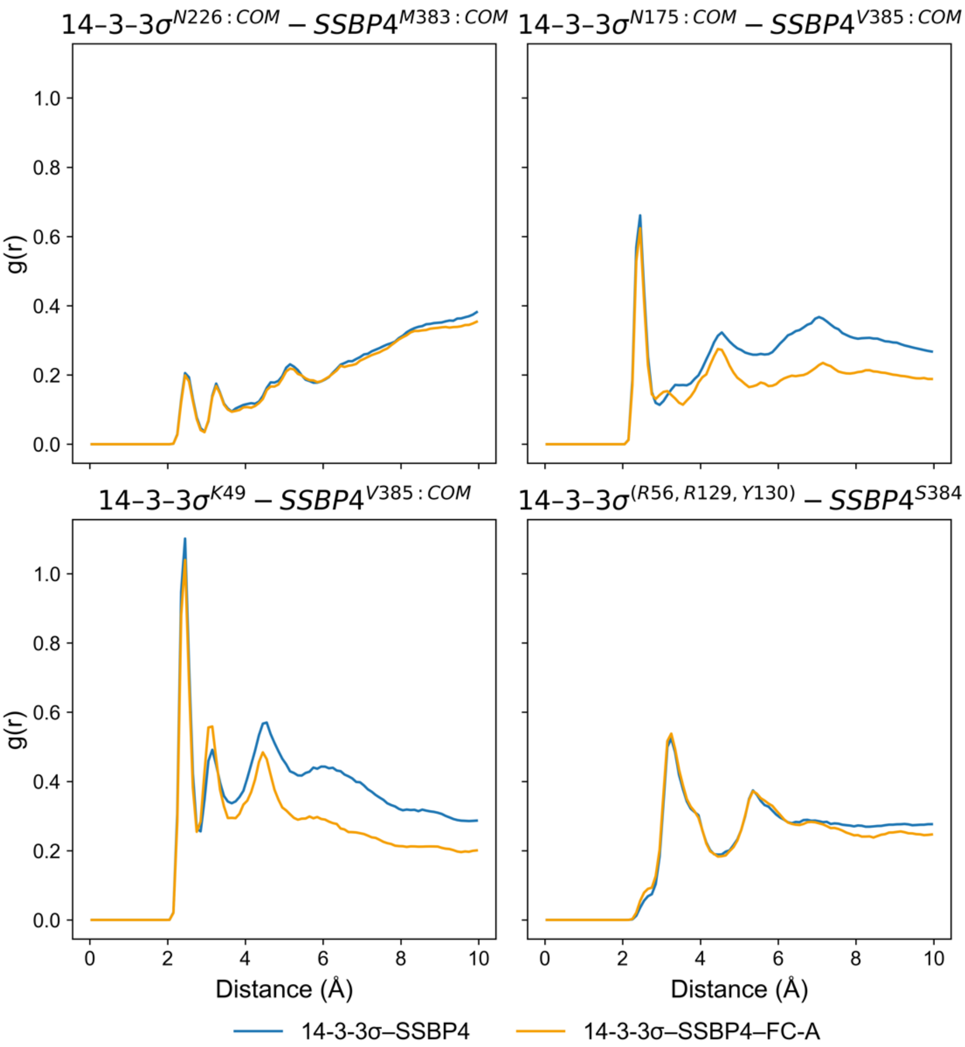

Figure S8. Radial distribution function of water around the atoms involved in the interactions considered for the 14-3-3σ–SSBP4 system. *The binary complex is depicted in marine and the ternary complex with FC-A is depicted in orange.*

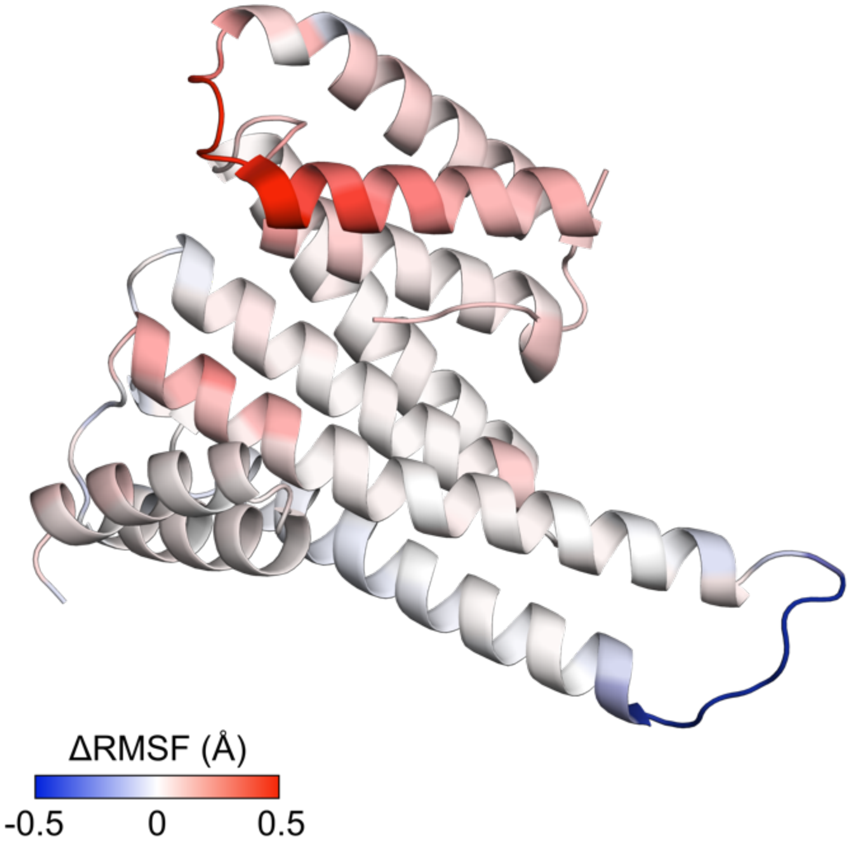

Figure S9. Flexibility of the helix H9 14-3-3σ–SSBP4 system depending on the presence or absence of FC-A*. RMSF difference between the non-FC-A and the holo structures along the sampling trajectories.*

Table S1. Average Pearson correlation coefficients of the distance values of the two interactions at the CRBN–SALL4 interface when the third interaction is pulled apart in SMD trajectories*. The average was obtained from the inverse of the average Fisher's transformed correlation coefficients from individual trajectories.*

Pulled interaction	Interactions	Pearson coefficient (r)			
		CRBN–SALL4	CRBN–SALL4–POM	CRBN–SALL4–LEN	CRBN–SALL4–THA
$CRBN^{N351}$–$SALL4^{S413}$	$CRBN^{H357}$–$SALL4^{V414}$	0.33	0.25	0.31	0.23
$CRBN^{N351}$–$SALL4^{S413}$	$CRBN^{W400}$–$SALL4^{C415}$	0.33	0.31	0.29	0.34
$CRBN^{H357}$–$SALL4^{V414}$	$CRBN^{N351}$–$SALL4^{S413}$	0.31	0.07	0.13	0.05
$CRBN^{H357}$–$SALL4^{V414}$	$CRBN^{W400}$–$SALL4^{C415}$	**0.55**	-0.02	0.05	0.02
$CRBN^{W400}$–$SALL4^{C415}$	$CRBN^{N351}$–$SALL4^{S413}$	0.14	0.14	0.16	0.22
$CRBN^{W400}$–$SALL4^{C415}$	$CRBN^{H357}$–$SALL4^{V414}$	0.16	0.00	0.18	0.17

Table S2. Average Pearson correlation coefficients of the distance values of the six interactions at the CA14–DB21 interface when the seventh interaction is pulled apart in SMD trajectories*. The average was obtained from the inverse of the average Fisher's transformed correlation coefficients from individual trajectories. The correlations in red indicate that one of the interactions of the pair has been discarded due to the high correlation with other distances.*

Pulled interaction	Interactions	Pearson coefficient (r)	
		CA14–DB21	CA14–DB21–CBD
CA14$^{D34:COM}$–DB21^{R46}	CA14^{Q58}–DB21^{F48}	0.02	0.08
CA14$^{D34:COM}$–DB21^{R46}	CA14^{P59}–DB21^{Y61}	0.30	0.06
CA14$^{D34:COM}$–DB21^{R46}	CA14^{K101}–DB21$^{E108:COM}$	0.16	-0.04
CA14$^{D34:COM}$–DB21^{R46}	CA14^{N106}–DB21^{E108} (1)	-0.05	-0.01
CA14$^{D34:COM}$–DB21^{R46}	CA14^{N106}–DB21^{E108} (2)	0.02	0.01
CA14$^{D34:COM}$–DB21^{R46}	CA14^{Q58}–DB21^{Y61}	0.10	0.08
CA14^{Q58}–DB21^{F48}	CA14$^{D34:COM}$–DB21^{R46}	0.08	0.29
CA14^{Q58}–DB21^{F48}	CA14^{P59}–DB21^{Y61}	0.38	0.16
CA14^{Q58}–DB21^{F48}	CA14^{K101}–DB21$^{E108:COM}$	0.03	-0.07
CA14^{Q58}–DB21^{F48}	CA14^{N106}–DB21^{E108} (1)	0.0	0.01
CA14^{Q58}–DB21^{F48}	CA14^{N106}–DB21^{E108} (2)	0.01	0.00
CA14^{Q58}–DB21^{F48}	CA14^{Q58}–DB21^{Y61}	**0.62**	0.46
CA14^{P59}–DB21^{Y61}	CA14$^{D34:COM}$–DB21^{R46}	0.09	0.20
CA14^{P59}–DB21^{Y61}	CA14^{Q58}–DB21^{F48}	0.39	0.41
CA14^{P59}–DB21^{Y61}	CA14^{K101}–DB21$^{E108:COM}$	-0.03	0.01
CA14^{P59}–DB21^{Y61}	CA14^{N106}–DB21^{E108} (1)	-0.00	-0.01
CA14^{P59}–DB21^{Y61}	CA14^{N106}–DB21^{E108} (2)	0.03	0.00
CA14^{P59}–DB21^{Y61}	CA14^{Q58}–DB21^{Y61}	0.42	0.40
CA14^{K101}–DB21$^{E108:COM}$	CA14$^{D34:COM}$–DB21^{R46}	0.41	0.45
CA14^{K101}–DB21$^{E108:COM}$	CA14^{Q58}–DB21^{F48}	0.15	0.03
CA14^{K101}–DB21$^{E108:COM}$	CA14^{P59}–DB21^{Y61}	0.30	0.01
CA14^{K101}–DB21$^{E108:COM}$	CA14^{N106}–DB21^{E108} (1)	0.02	0.05
CA14^{K101}–DB21$^{E108:COM}$	CA14^{N106}–DB21^{E108} (2)	0.24	0.21
CA14^{K101}–DB21$^{E108:COM}$	CA14^{Q58}–DB21^{Y61}	0.21	0.03
CA14^{N106}–DB21^{E108} (1)	CA14$^{D34:COM}$–DB21^{R46}	0.04	0.09
CA14^{N106}–DB21^{E108} (1)	CA14^{Q58}–DB21^{F48}	0.22	0.07
CA14^{N106}–DB21^{E108} (1)	CA14^{P59}–DB21^{Y61}	0.22	0.01
CA14^{N106}–DB21^{E108} (1)	CA14^{K101}–DB21$^{E108:COM}$	0.04	0.07
CA14^{N106}–DB21^{E108} (1)	CA14^{N106}–DB21^{E108} (2)	0.14	0.05
CA14^{N106}–DB21^{E108} (1)	CA14^{Q58}–DB21^{Y61}	0.20	-0.01
CA14^{N106}–DB21^{E108} (2)	CA14$^{D34:COM}$–DB21^{R46}	-0.07	0.08
CA14^{N106}–DB21^{E108} (2)	CA14^{Q58}–DB21^{F48}	0.27	0.12
CA14^{N106}–DB21^{E108} (2)	CA14^{P59}–DB21^{Y61}	0.35	0.00
CA14^{N106}–DB21^{E108} (2)	CA14^{K101}–DB21$^{E108:COM}$	0.10	0.10
CA14^{N106}–DB21^{E108} (2)	CA14^{N106}–DB21^{E108} (1)	0.07	0.16
CA14^{N106}–DB21^{E108} (2)	CA14^{Q58}–DB21^{Y61}	0.23	0.07
CA14^{Q58}–DB21^{Y61}	CA14$^{D34:COM}$–DB21^{R46}	0.04	0.31
CA14^{Q58}–DB21^{Y61}	CA14^{Q58}–DB21^{F48}	**0.58**	**0.55**
CA14^{Q58}–DB21^{Y61}	CA14^{P59}–DB21^{Y61}	**0.65**	0.13
CA14^{Q58}–DB21^{Y61}	CA14^{K101}–DB21$^{E108:COM}$	0.08	0.02
CA14^{Q58}–DB21^{Y61}	CA14^{N106}–DB21^{E108} (1)	0.01	0.01
CA14^{Q58}–DB21^{Y61}	CA14^{N106}–DB21^{E108} (2)	0.03	0.03

Table S3. Average Pearson correlation coefficients of the distance values of the five interactions at the 14-3-3σ–SSBP4 interface when the sixth interaction is pulled apart in SMD trajectories*. The average was obtained from the inverse of the average Fisher's transformed correlation coefficients from individual trajectories. The correlations in red indicate that one of the interactions of the pair has been discarded due to the high correlation with other distances.*

Pulled interaction	Interactions	Pearson coefficient (r)	
		14-3-3σ–SSBP4	14-3-3σ–SSBP4–FC-A
14-3-3σ^{W230}–SSBP4^{T382}	14-3-3σ$^{N226:COM}$–SSBP4$^{M383:COM}$	0.25	0.17
14-3-3σ^{W230}–SSBP4^{T382}	14-3-3σ$^{(R56,R129,Y130)}$–SSBP4^{S384}	0.06	0.03
14-3-3σ^{W230}–SSBP4^{T382}	14-3-3σ^{K122}–SSBP4$^{V385:COM}$	0.02	-0.04
14-3-3σ^{W230}–SSBP4^{T382}	14-3-3σ^{K49}–SSBP4$^{V385:COM}$	-0.04	0.06
14-3-3σ^{W230}–SSBP4^{T382}	14-3-3σ$^{N175:COM}$–SSBP4$^{V385:COM}$	0.01	0.05
14-3-3σ$^{N226:COM}$–SSBP4$^{M383:COM}$	14-3-3σ^{W230}–SSBP4^{T382}	**0.71**	**0.66**
14-3-3σ$^{N226:COM}$–SSBP4$^{M383:COM}$	14-3-3σ$^{(R56,R129,Y130)}$–SSBP4^{S384}	-0.04	-0.08
14-3-3σ$^{N226:COM}$–SSBP4$^{M383:COM}$	14-3-3σ^{K122}–SSBP4$^{V385:COM}$	-0.15	-0.08
14-3-3σ$^{N226:COM}$–SSBP4$^{M383:COM}$	14-3-3σ^{K49}–SSBP4$^{V385:COM}$	0.18	0.05
14-3-3σ$^{N226:COM}$–SSBP4$^{M383:COM}$	14-3-3σ$^{N175:COM}$–SSBP4$^{V385:COM}$	0.08	0.07
14-3-3σ$^{(R56,R129,Y130)}$–SSBP4^{S384}	14-3-3σ^{W230}–SSBP4^{T382}	**0.6**	**0.61**
14-3-3σ$^{(R56,R129,Y130)}$–SSBP4^{S384}	14-3-3σ$^{N226:COM}$–SSBP4$^{M383:COM}$	0.29	0.32
14-3-3σ$^{(R56,R129,Y130)}$–SSBP4^{S384}	14-3-3σ^{K122}–SSBP4$^{V385:COM}$	-0.17	-0.15
14-3-3σ$^{(R56,R129,Y130)}$–SSBP4^{S384}	14-3-3σ^{K49}–SSBP4$^{V385:COM}$	**0.51**	0.41
14-3-3σ$^{(R56,R129,Y130)}$–SSBP4^{S384}	14-3-3σ$^{N175:COM}$–SSBP4$^{V385:COM}$	0.17	-0.15
14-3-3σ^{K122}–SSBP4$^{V385:COM}$	14-3-3σ^{W230}–SSBP4^{T382}	-0.15	-0.12
14-3-3σ^{K122}–SSBP4$^{V385:COM}$	14-3-3σ$^{N226:COM}$–SSBP4$^{M383:COM}$	-0.04	-0.07
14-3-3σ^{K122}–SSBP4$^{V385:COM}$	14-3-3σ$^{(R56,R129,Y130)}$–SSBP4^{S384}	0.03	0.11
14-3-3σ^{K122}–SSBP4$^{V385:COM}$	14-3-3σ^{K49}–SSBP4$^{V385:COM}$	-0.36	-0.37
14-3-3σ^{K122}–SSBP4$^{V385:COM}$	14-3-3σ$^{N175:COM}$–SSBP4$^{V385:COM}$	0.48	**0.52**
14-3-3σ^{K49}–SSBP4$^{V385:COM}$	14-3-3σ^{W230}–SSBP4^{T382}	0.25	0.05
14-3-3σ^{K49}–SSBP4$^{V385:COM}$	14-3-3σ$^{N226:COM}$–SSBP4$^{M383:COM}$	0.12	0.02
14-3-3σ^{K49}–SSBP4$^{V385:COM}$	14-3-3σ$^{(R56,R129,Y130)}$–SSBP4^{S384}	0.03	0.07
14-3-3σ^{K49}–SSBP4$^{V385:COM}$	14-3-3σ^{K122}–SSBP4$^{V385:COM}$	-0.3	-0.11
14-3-3σ^{K49}–SSBP4$^{V385:COM}$	14-3-3σ$^{N175:COM}$–SSBP4$^{V385:COM}$	0.1	-0.03
14-3-3σ$^{N175:COM}$–SSBP4$^{V385:COM}$	14-3-3σ^{W230}–SSBP4^{T382}	-0.03	-0.05
14-3-3σ$^{N175:COM}$–SSBP4$^{V385:COM}$	14-3-3σ$^{N226:COM}$–SSBP4$^{M383:COM}$	0.04	0
14-3-3σ$^{N175:COM}$–SSBP4$^{V385:COM}$	14-3-3σ$^{(R56,R129,Y130)}$–SSBP4^{S384}	0.08	0.12
14-3-3σ$^{N175:COM}$–SSBP4$^{V385:COM}$	14-3-3σ^{K122}–SSBP4$^{V385:COM}$	**0.9**	**0.89**
14-3-3σ$^{N175:COM}$–SSBP4$^{V385:COM}$	14-3-3σ^{K49}–SSBP4$^{V385:COM}$	-0.11	-0.1

Available Datasets

Dataset S1: *An open data repository is accessible at:*

https://bitbucket.org/jjuarez84/mg_hbonds/src/'main'/

It contains:

- Topologies and equilibrated coordinate files for each of the SMD systems.

- Sample AMBER input files.

- Unprocessed W profiles obtained from SMD trajectories that were used to build the PMF_{HB_break} profiles.

- PDB files of the output of the MD simulations described in the manuscript.
